# Ecomorphological convergence in the walking leg dactyli of two clades of ascidian‐ and mollusc‐associated shrimps (Decapoda: Caridea: Palaemonidae)

**DOI:** 10.1002/ece3.10768

**Published:** 2023-12-19

**Authors:** Werner de Gier, Pepijn Helleman, Jurriaan van den Oever, Charles H. J. M. Fransen

**Affiliations:** ^1^ Naturalis Biodiversity Center Leiden The Netherlands; ^2^ Groningen Institute for Evolutionary Life Sciences University of Groningen Groningen The Netherlands; ^3^ Institute of Biology Leiden Leiden University Leiden The Netherlands

**Keywords:** adaptive radiation, morphometrics, phylomorphospace, SEM‐photography, symbiosis

## Abstract

Symbiotic species, living within or on the surface of host organisms, may evolve a wide range of adaptations as a result of various selection pressures, host specificity of the symbiont and the nature of the symbiosis. In tropical marine coral reef ecosystems, palaemonid shrimps (Crustacea: Decapoda: Caridea) live in association with at least five different invertebrate phyla. Host switches between (distantly) related host groups, and the thereby associated selection pressures were found to play a major role in the diversification of these shrimp lineages, giving rise to various host‐specific adaptations. Two lineages of palaemonid shrimp, which have switched from an ectosymbiotic association towards endosymbiosis, are studied for their morphological diversification and possible convergence. Special attention is given to the between‐phyla host switches involving ascidian and bivalve hosts, which are characteristic for these lineages. Using landmark‐based (phylo)morphospace analyses and Scanning Electron Microscopy, the walking leg dactylus shape and the microstructures on these dactyli are studied. No specific bivalve‐ or ascidian‐associated morphotypes were found, but morphological convergence in dactylus morphology was found in various species within the two studied clades with similar host groups. In addition, multiple lineages of bivalve‐associated species appear to be morphologically diverging more than their ascidian‐associated relatives, with ‘intermediate’ morphotypes found near host‐switching events.

## Graphical abstract

The walking leg dactyli of two clades of endosymbiotic shrimp species, living in ascidians and bivalves, are analysed using (phylo)morphometric methods. The two clades show between‐ and within‐clade morphological convergence in both the overall shape of the dactyli, as well as microstructures which can only be seen with SEM‐photography. Bivalve‐associated species showcase more shape variation than their ascidian‐associated relatives, and this seems to be linked to host‐switching events in their phylogeny.

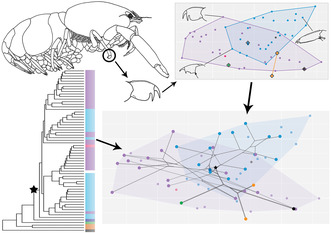

## 1 INTRODUCTION

Biodiversity, and its variation in morphology, is shaped by various types of selection pressures (e.g. new dietary habits, predators or environmental factors; Burin et al., 2016; Edelaar et al., 2017; Losos & Ricklefs, 2009), which result in evolutionary processes like speciation and extinction (Jablonski, 2008). In most classical examples, these selection pressures are the result of a species colonizing a newly available habitat and filling up a niche (e.g. adaptive radiation; Schluter, 2000). Although schoolbook examples mostly focus on macroevolutionary processes, such as island biogeography, habitat shifts might also occur on a much smaller, sympatric scale. For instance, in symbiotic relationships, a shift of a symbiont from one host species to another might also result in new selection pressures for the symbiont. Depending on the strength of the selection pressures, the host specificity of the symbiont, and the nature of the symbiosis (obligatory or facultative commensalism, mutualism or parasitism), new species might rapidly arise from these host switches (Doña et al., 2018). Moreover, depending on the variation in host types due to host‐switching events between distantly related hosts, a wide range of morphological adaptations might evolve in the symbiont species, resulting in a relatively high degree of morphological variation in these clades (Ghalambor et al., 2007; Joy, 2013; Kise et al., 2023; Munday et al., 2004; Sapp, 1994). Convergent evolution may also play a role, if similar selection pressures are at play in different clades of symbionts, resulting in the same, or similar (analogous or homologous) adaptations (Goto et al., 2012; Horká et al., 2018; Kise et al., 2023; Li et al., 2018; Pérez‐Losada et al., 2009; Poulin & Randhawa, 2015).

A group of animals where this rapid evolution of adaptive features seems to have occurred is a clade of marine symbiotic shrimp species in the family Palaemonidae (Crustacea: Decapoda: Caridea). It is estimated that about 70% of the marine species in this family have established some form of symbiotic interaction (De Grave, 2001). Most species of symbiotic palaemonid shrimps are involved in protective symbioses (Chow et al., 2021), meaning that they live inside the body cavity of their host (endosymbiotic, as used in Baeza, 2015) or on the surface of their host (ectosymbiotic), supplying a shrimp with dietary needs or a certain level of protection through either providing shelter or camouflaging possibilities. Most of these associations have evolved with fellow coral reef inhabitants, including species of the phyla Porifera, Cnidaria, Mollusca, Echinodermata and Chordata (Bruce, 1995; Chow et al., 2021; Horká et al., 2016). In addition, so‐called ‘inquilinistic or inquiline forms’ of palaemonid shrimps have evolved to share a burrow with pistol shrimps (Caridea: Alpheidae), opisthognath fish or echiurids (Frolová et al., 2022). This above‐mentioned host range is presumably an incomplete record, as many other symbiotic interactions have not been identified yet, and more associations are still being discovered (Anker & De Grave, 2021; de Gier & Fransen, 2018; Fransen et al., 2021, 2022; Komai et al., 2023; Rauch et al., 2019). Several studies have demonstrated interphylum host switching to be important in the past diversification of the family (Davis et al., 2018; Gan et al., 2015; Horká et al., 2016, 2018; Kou et al., 2013, 2015). Host‐switching events, together with coadaptation and cospeciation, have been argued to be the main drivers of diversification within this group (Horká et al., 2016).

**FIGURE 1:**
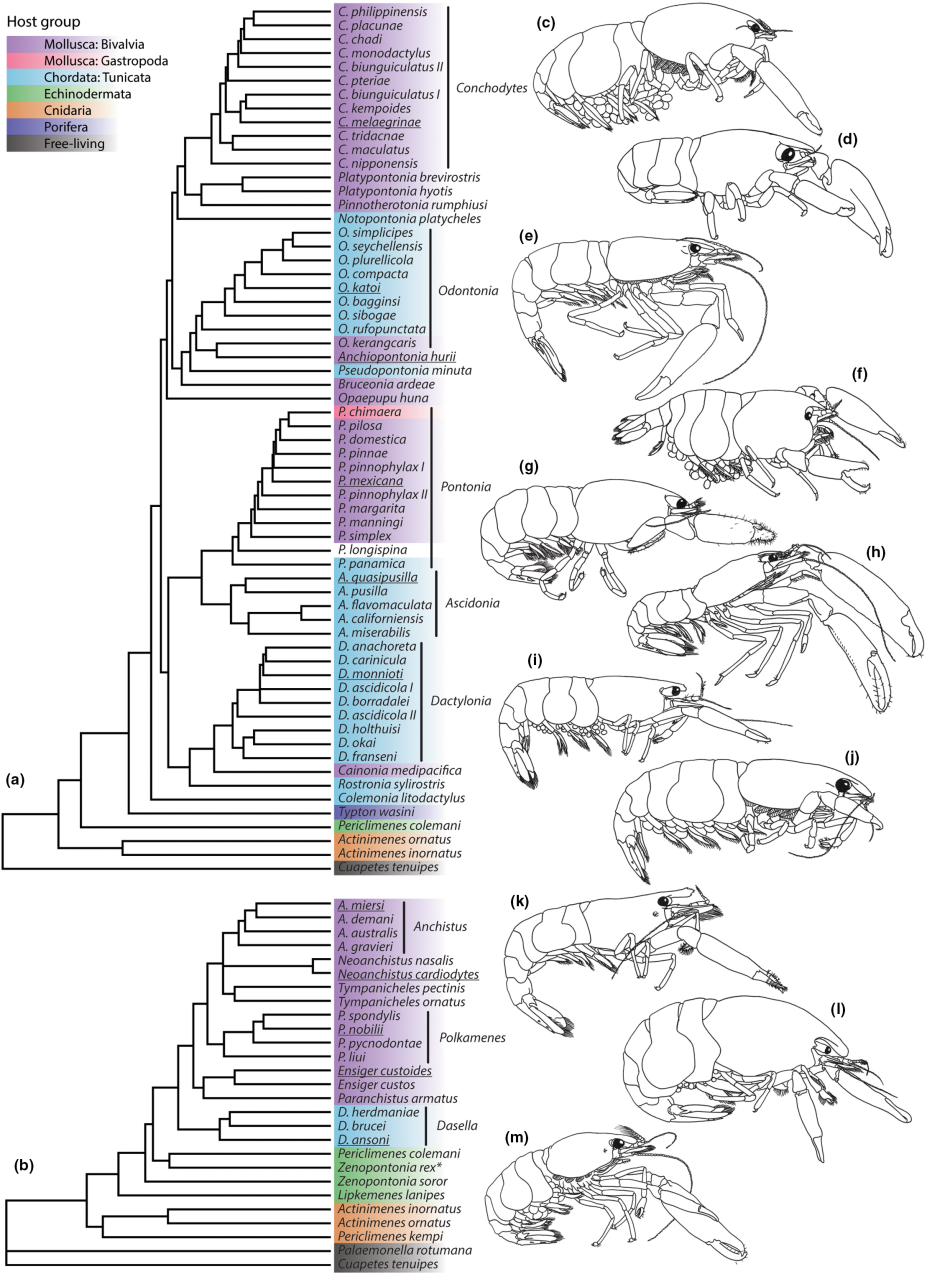
Phylogeny and morphological variation in the ‘*Conchodytes* clade’ (a) and the ‘*Anchistus* clade’ (b). Ultrametric phylogeny reconstructions based on TE‐analyses from de Gier et al. (2022) (a) and de Gier and Fransen (2023) (b). (c–m) Lateral views of a number of representatives from the two clades. Representatives are underlined in the phylogeny trees: (c) *Conchodytes meleagrinae* Peters, 1852—after Fransen & Reijen (2013); (d) *Odontonia katoi* (Kubo, 1940)—after Hayashi (2003); (e) *Anchiopontonia hurii* (Holthuis, 1981)—after Bruce (1992); (f) *Pontonia mexicana* Guérin‐Méneville, 1855—after an illustration by Alberto Guerra, for BioDiversidad Marina de Yucatán (BDMY); (g) *Ascidonia quasipusilla* (Chace, 1972)—new illustration of RMNH.CRUS.D.51678; (h) *Dactylonia monnioti* (Bruce, 1990)—after Bruce (1990); (i) *Anchistus miersi* (De Man, 1888)—after de Gier and Fransen (2023); (j) *Neoanchistus cardiodytes* Bruce, 1975—after Bruce (1975); (k) *Polkamenes nobilii* (Holthuis, 1952)—after de Gier and Fransen (2023); (l) *Ensiger custoides* (Bruce, 1977)—after de Gier and Fransen (2023); (m) *Dasella ansoni* Bruce, 1983—after Bruce (1983). Shrimp dimensions not to scale. Colours indicate host groups (see legend), with *Zenopontonia rex* (Kemp, 1922) being ectosymbiotic with holothurians and nudibranch gastropods (indicated by an asterisk (*)).

Host‐switching events seem to have occurred a few times in ectosymbiotic clades within the Palaemonidae, mainly between echinoderm and cnidarian hosts (Horká et al., 2016). In endosymbiotic lineages, like Clades 5 and 6 of Horká et al. (2016; see below), these events are hypothesized to have happened much more frequent (de Gier et al., 2022). The endosymbiotic mollusc‐ and ascidian‐associated palaemonid species are generally grouped in two clades (Figure 1; de Gier et al., 2022; de Gier & Fransen, 2023), from hereon called the ‘*Conchodytes* clade’ and the ‘*Anchistus* clade’. The first clade includes the larger genera *Ascidonia* Fransen, 2002, *Conchodytes* Peters, 1852, *Dactylonia* Fransen, 2002, *Odontonia* Fransen, 2002 and *Pontonia* Latreille, 1829, in addition to some minor, often monotypic genera (de Gier et al., 2022). The second clade includes the genera *Anchistus* Borradaile, 1898; *Dasella* Labour, 1945; *Ensiger* Borradaile, 1915; *Neoanchistus* Bruce, 1975; *Paranchistus* Holthuis 1952; *Polkamenes* de Gier & Fransen, 2023; and *Tympanicheles* de Gier & Fransen, 2023 (de Gier & Fransen, 2023). The two clades are not directly related, and the placement of the larger clades within the Palaemonidae is still a point of discussion: in the study by Horká et al. (2016) the ‘*Conchodytes* clade’ makes up its own clade (Clade 6), while the ‘*Anchistus* clade’ is part of a larger sister clade including ectosymbiotic species (Clade 5). In a more recent study by Chow et al. (2021), the ‘*Conchodytes* clade’ makes up part of a large clade including even some free‐living deep‐sea species (Clade IIIC), while the ‘*Anchistus* clade’ can be found in a different branch together with most of the outgroups in this study (Clade IIIH). The endosymbiotic lifestyle of these clades seems to be mirrored by their morphological variation: lots of species exhibit morphological adaptations that seem to be linked to an endosymbiotic lifestyle inside a soft‐bodied host. Although the two clades are unrelated, these adaptations can, to some extent, be found in both species groups. The adaptations often include: (A) adaptive colouration, thereby enhancing camouflage; (B) a cylindrical‐shaped body to fit in the internal cavity of the host (Figure 1); (C) a reduction in the size and number of frontal, dorsal and lateral protrusions from the body, thereby aiding movement inside the host's cavity (see examples in Figure 1); (D) altered eye morphology; and (E) the presence of (micro‐)structures on the ambulatory dactyli (such as denticles, hooked teeth and microsetae) (Bruce, 1972, 1976, 1980, 1995; de Gier et al., 2022; Dobson et al., 2014, 2016; Fransen, 1994). In contrast, most free‐living species of palaemonid shrimp carry a more ornamented rostrum and carapace (Fransen, 1994), and have less complex dactyli (Chow et al., 2021). A number of adaptations mentioned above (B, C and E) are also found in other groups of endosymbiotic crustaceans, such as pea and gall crabs (Decapoda: Brachyura: resp. Pinnotheridae and Cryptochiridae) (de Gier & Becker, 2020; Vehof et al., 2016), and commensal or parasitic copepods (Gotto, 2004).

The above‐mentioned adaptations may be linked to an endosymbiotic lifestyle, but most ecomorphological hypotheses are based on short remarks on a few selected species in taxonomic and ecological literature (Bruce, 1976, 1995; Fransen, 1994, 2002). We now have the means (an extensive voucher collection, as well as various new figuration and analysis methods) to properly study these morphological adaptations in an evolutionary context. In this study, we focus on the third pereiopod dactyli, both by mapping the diversity of the overall shapes as well as the microstructures found on the unguis and corpus (see Figure S1). The extreme (family‐wide) variation in walking leg dactylus morphology is used in taxonomic and phylogenetic context to identify new species, delimit species groups, and to map the evolution of the marine Palaemonidae (Bruce, 2013; de Gier & Fransen, 2018; Fransen, 2002). Compared to other morphological structures which are often used in taxonomic contexts (e.g. the rostrum and telson spines; see for example de Gier & Fransen, 2023), the walking leg dactyli showcase almost no intraspecific variance (Chow et al., 2021). The walking legs are thought to be one of the first features to adapt to new surroundings, and are therefore prime candidates for ecomorphological studies on the taxa (Fransen, 2002).

As of today, there is no information on the host specificity of the above‐mentioned adaptations; although hosts from different phyla may share a similar protective microhabitat (Horká et al., 2016), we suspect some morphological adaptations to be ascidian‐ or mollusc‐ specific in these shrimp clades. Additionally, some morphological adaptations might be present in species which are restricted to only one family of bivalve or ascidian hosts. We also expect convergent evolution to play a major role in the morphological variation within these clades when switching back to a similar host, and will study these within‐clade patterns in detail. Although the clades also show some similarities, the questionable placement of the two clades within the family‐wide phylogeny tree did not allow for a possible between‐clade convergence analysis. This study combines phylogenetic information from previous literature (de Gier et al., 2022; de Gier & Fransen, 2023) with 2D morphometric analyses and microscopic imaging methods (scanning electron microscope; SEM), and attempts to elucidate the plasticity in shrimp dactylus morphology in relation to the various known host‐switches in the evolutionary history of the selected clades.

## 2 MATERIALS AND METHODS

### 2.1 Species selection and data acquisition

The ‘*Anchistus* clade’ and ‘*Conchodytes* clade’, consisting of respectively 18 and 55 currently known species (de Gier et al., 2022; de Gier & Fransen, 2023), were used as ingroups. We included a wide range of endo‐ and ectosymbiotic shrimps that are related to the studied clades, as well as two species with a free‐living lifestyle, as outgroups. The outgroup selection is the same as in the studies by de Gier et al. (2022) and de Gier and Fransen (2023). Dactylus shape data were acquired from taxonomic literature. No notable intraspecific variation in the third pereiopod dactylus shape has been recorded for the included species, so one specimen for each species was used (as in de Gier, 2023). For the newly acquired SEM images, collection material was used that was obtained during historical and recent expeditions and has been deposited in the decapod collection of Naturalis Biodiversity Center (previously Rijksmuseum van Natuurlijke Historie; RMNH.CRUS.D). These samples were collected using standard collection procedures in the field (mainly SCUBA diving) and stored in 70% ethanol. The materials used for the SEM study, as well as the references used during the literature search, are listed in Table S1.

### 2.2 2D morphometrics

A total of 18 landmarks (7 stationary landmarks, 11 semi‐landmarks) were placed on 2D lateral images of the third pereiopod dactylus of all studied species (Table S1). Homologous landmarks were chosen based on the full dataset; where comparable anatomical features could not be landmarked due to their absence, the landmarks fall on the same anatomical point (Gómez‐Robles et al., 2011). The landmarks are listed and annotated in the supplementary datafile (Figure S1).

Landmark data were gathered in tpsDig2 (v. 2.31) (Rohlf, 2017) and analysed using R v. 4.2.1 and RStudio v. 2022.07.0 (R Core Team, 2022; RStudio Team, 2022), using the packages *geomorph* v. 4.0.4 and *ggplot2* v. 3.3.6 (Adams et al., 2022; Baken et al., 2021; Wickham, 2016). A generalised Procrustes analysis (GPA) was performed to scale, transform and rotate all images for morphospace analyses. In this way, scale was set to ‘uniform’ and centroid size was not taken into account. Species were coloured in the morphospaces based on their host association (similar to de Gier, 2023). Morphospaces with all species annotated in the plot can be found in the supplementary datafile (Figures S2 and S3; numbers in Table S1). A morphospace with both clades included was also built to check for resemblance between the two clades. The resulting plots are added to the supplementary datafile (Figure S4). A Procrustes (M)ANOVA with a residual randomization permutation procedure (1000 permutations, RRPP) (Collyer & Adams, 2021) was performed to detect significant effects of the host associations on the placement of the species in the morphospaces, and a pairwise test was performed to find significant differences in the mean shape data in relation to host specificity. Distance between the mean shapes (*d*), upper confidence level (UCL (95%)) and *p*‐values associated with the mean shape distances (Pr > *d*) are given (de Gier, 2023).

### 2.3 Molecular phylogenies and phylomorphospace‐related analyses

Molecular phylogeny reconstructions of the two clades were used to project the evolutionary history of the species on the morphospaces, resulting in phylomorphospaces (Stayton, 2015). The total evidence (TE) phylogeny trees from the studies by de Gier et al. (2022) and de Gier and Fransen (2023) were built using both molecular and morphological character states. For the ‘*Conchodytes* clade’, the analyses included two mitochondrial (*COI* and *16S* rRNA) and two nuclear genes (*histone H3* and *18S* rRNA), while for the ‘*Anchistus* clade’, *18S* and *H3* were omitted due to their limited phylogenetic signalling. For more specifics, see de Gier et al. (2022) and de Gier and Fransen (2023). Since the TE trees are partially based on morphological characters found in the walking leg dactyli, the phylogenies were trimmed to include only specimens for which molecular data were available. This excludes any dependency of the tree topologies on the morphological character state analysis. Only one specimen of those species was included for each branch, with the exception of species with a polyphyletic placement (de Gier et al., 2022).

Phylomorphospaces were built in R using the packages phytools, geiger and ape (see above). The similarity‐based measures C_1_ to C_4_ with corresponding P‐values and 1000 replicates (de Gier, 2023; Stayton, 2015) were calculated using the R package *convevol* v. 1.3 (Stayton, 2018). The convergence measures and their calculations are described by Stayton (2015). For the calculations of the C‐values, PC‐values were used from PC1 to PC3 (81% and 80% of the explained data for the ‘*Conchodytes’* and ‘*Anchistus* clade’, respectively). Species pairs with a short distance in the morphospace were selected for both clades: five pairs within the ‘*Conchodytes* clade’ (two between genera with the same host group; one within a genus with the same host group; and two between two genera with a different host group) and three pairs in the ‘*Anchistus* clade’ (two between two genera with the same host group; and one between two genera with different host groups).

Two phylogenetically informed ANOVAs (phylogenetic generalized least squares; PGLS) were performed in a similar way as the one described by de Gier (2023). This was done to investigate the impact of host choice on the shape variation in the 2D data of the two studied clades, while controlling for independence of the residuals from the phylogeny (Adams & Collyer, 2018; Mundry, 2014). This was done for 37 species in the ‘*Conchodytes* clade’, including five outgroup species and two polyphyletic species, and 18 species in the ‘*Anchistus* clade’ (including nine outgroup species), by using the procD.pgls() command in *geomorph*, with Pagel's lambda (λ) set at 1.0 (a high phylogenetic signal—Brownian motion model; Pagel, 1999). For comparison, regular Procrustes ANOVA regression was performed on these subsets of the data. This was done to see if the exclusion of the phylogenetic framework had any effect. In all analyses, a RRPP‐approach was used with 1000 permutations.

### 2.4 SEM study

A total of 49 specimens were sampled for a SEM study; 41 ingroup species (including one duplicate—*Polkamenes pycnodontae* (Bruce, 1978) from an aberrant host (de Gier & Fransen, 2023)) and eight outgroup species (Table S1). Specimens are studied and prepared under a dissecting microscope (Zeiss Discovery v.8). From every specimen, the right third pereiopod was subsampled using dissecting tweezers, preferably from an ovigerous female specimen. The dactyli were cleaned with a fine brush under the dissecting microscope to remove any host tissue and sediment material. The pereiopods were then dehydrated in an ethanol series: 2 × 15 min in 80% ethanol, followed by 2 × 15 min in 96% ethanol, followed by 2 × 30 min in 100% acetone. Afterwards, the samples were dried using critical point drying (CPD) methods with a Leica EM CPD300 (Naturalis Biodiversity Center, Imaging facilities), according to the protocol used by de Gier and Fransen (2018). Each pereiopod was placed on a single stub using double‐sided tape and prepared for the SEM by coating the samples with a 20‐nm Pt/Pd coating using a Quorum Q150T S.

The dactyli were photographed using a JEOL JSM 6480LV SEM (Naturalis Biodiversity Center, Imaging facilities). The SEM gives the possibility to take very detailed photographs of microstructures on the dactyli, which were scored as four different morphological character states. The used character states can be found in the supplementary datafile (below Figure S1). Species of which the microstructures were tentatively placed in a character state were annotated with an asterisk in the results (e.g. the small, but still visible accessory tooth of the species in *Zenopontonia* Bruce, 1975 are annotated as 0* in the fourth character list). The entire dactylus, the unguis and the accessory spine (if present) were photographed for every sample. Additionally, minute microstructures were photographed in more detail if present. Plates were edited, and backgrounds were deleted in Adobe Photoshop. All SEM‐captures, with insets of relevant details, can be found in the supplementary datafile (Figures S5–S11).

## 3 RESULTS

### 3.1 Landmark analyses and morphospaces

**FIGURE 2:**
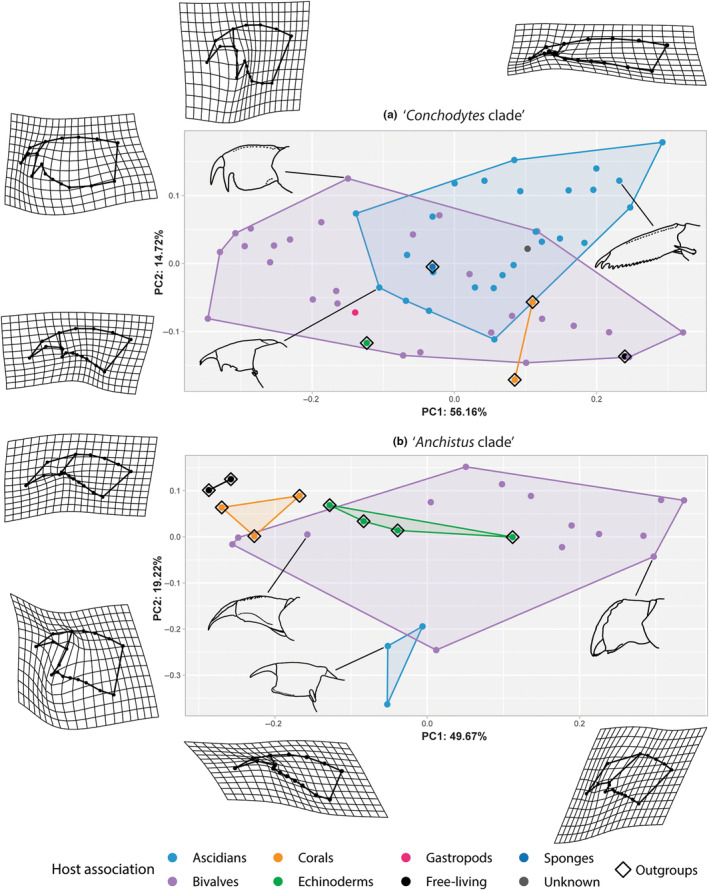
Morphospaces of the third pereiopod dactylus shape variation, resulting from the generalized Procrustes analysis, including warps of the maximum and minimum shape differences possible along the first two PC axes, relative to the mean shape of the morphospace. (a) ‘*Conchodytes* clade’ (including five outgoups), (b) ‘*Anchistus* clade’ (including nine outgroups). Colours of convex hulls and points indicate host associations, and three representatives are illustrated for each clade: (a) *Conchodytes meleagrinae* Peters, 1852 (top left) (after Fransen & Reijnen, 2013), *Odontonia compacta* (Bruce, 1996) (bottom left), *Dactylonia franseni* Bruce, 2003 (middle right) (both after Fransen, 2002); (b) *Tympanicheles ornatus* (Holthuis, 1952) (top left); *Dasella herdmaniae* (Lebour, 1938) (bottom left), *Anchistus demani* Kemp, 1922 (middle right); all after de Gier and Fransen (2023). Diamonds indicate outgroup species. Annotations for each species are given in Figure S1.

In the morphospace of the ‘*Conchodytes* clade’, the first two PC axes explain 72.88%, with 99% being explained in the first 14 PCs, out of 32 dimensions (Figure 2a). The first two PC axes of the ‘*Anchistus* clade’ explain 68.89% of the data, with 99% being explained in the first 10 PCs, out of 26 dimensions (Figure 2b).

The variation of the dactyli shapes in the ‘*Conchodytes* clade’ shows almost no differences in the unguis morphology, while the shape of the corpus, including the accessory tooth, varies the most (Figure 2a). This can be seen in the first axis of the morphospace, ranging from an almost round corpus with a long accessory tooth (PC1_min_) to an elongated corpus shape with almost no accessory tooth (PC1_max_; see *Dactylonia franseni* Bruce, 2003; Figure 2a). The second axis shows a variation in the curvature of the ventral side of the corpus, ranging from a curved corpus with a short accessory tooth (PC2_min_), to an inflated corpus with a long tooth (PC2_max_; see *Conchodytes meleagrinae*; Figure 2a, top left). Although the outgroups are scattered within the two major convex hulls of the bivalve‐ and ascidian‐associated species (Figure 2a), these two latter groups seem to be only partially overlapping. Six bivalve‐associated species (*Anchiopontonia hurii* (Holthuis, 1981); *Cainonia medipacifica* (Edmondson, 1935); *Conchodytes monodactylus* Holthuis, 1952; *Pontonia mexicana* Guérin‐Méneville, 1855; *Pontonia pinnae* Lockington, 1878; and *Pontonia pinnophylax* (Otto, 1821)) and one species with an unknown host (*Pontonia longispina* Holthuis, 1951) overlap with the ascidian‐associated convex hull.

The species within the ‘*Anchistus* clade’ also show some variation in the shape of the corpus, but in contrast with the species above, the unguis also displays a large degree of variation (Figure 2b). The first displayed axis shows a morphological gradient, ranging from an elongated corpus, with a minute accessory tooth and a sharp, thin unguis (PC1_min_), to a more rounded corpus with a large and often broad (de Gier & Fransen, 2023) unguis (PC1_max_). The unguis might even be rounded at the apex, as is seen in *Anchistus demani* Kemp, 1922 (Figure 2b). On the second axis, the morphological variation ranges from a wide, almost square corpus, with a broad outgrowth between the ventral border of the unguis and the accessory tooth (PC2_min_; see *Dasella* in Figure 2b), to a dactylus with an elongated corpus and unguis, with the accessory tooth being absent (PC2_max_). This last shape, seen in three bivalve‐associated species (both species of *Ensiger*, and *Tympanicheles ornatus* (Holthuis, 1952); Figure 2b) is shared with the free‐living and coral‐associated outgroups. The chosen outgroups keep to one side of the plot, except for the echinoderm‐associated species, partially overlapping with the bivalve‐associated species. Only the ascidian‐associated *Dasella brucei* Berggren, 1990 overlaps with the convex hull of the bivalve‐associated species (Figure 2b).

When combining the two datasets in one morphospace (Figure S4, numbers in Table S1), it can be seen that the dactyli of some species within the ‘*Anchistus* clade’ are grouped together with the species in the ‘*Conchodytes* clade’. For example, *Paranchistus armatus* (H. Milne Edwards, 1837) groups together with some of the bivalve‐associated species in *Conchodytes*. The species within *Dasella*, while extending the range of the ascidian‐associated convex hull, statistically resemble *Odontonia bagginsi* de Gier & Fransen, 2018, following the morphospace. Moreover, almost all bivalve‐associated species within the ‘*Anchistus* clade’ group together in PC2_min_ (Figure S4a), showing almost no resemblance to the other clade in the shape of their dactyli.

**FIGURE 3:**
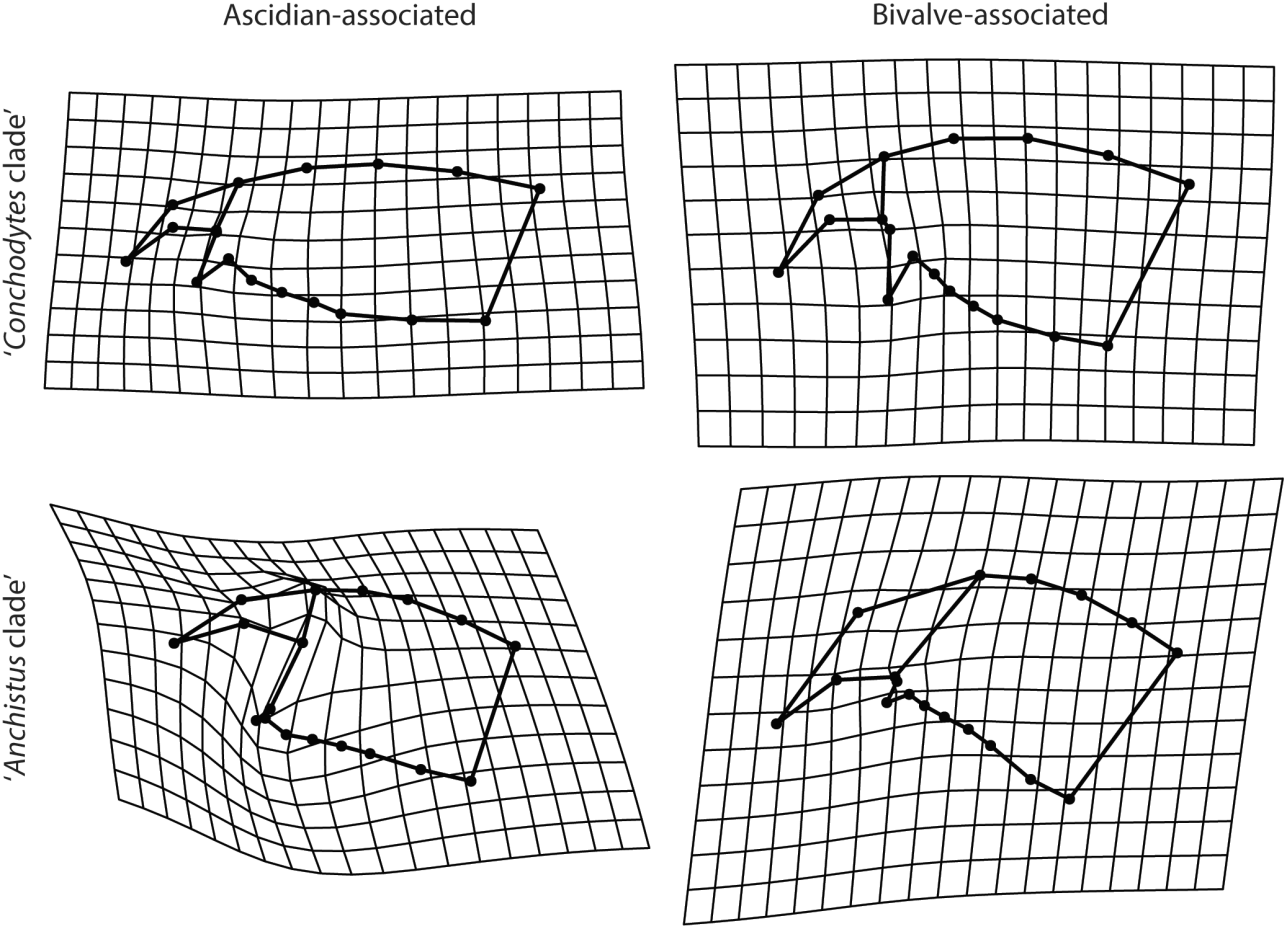
Mean dactylus shape warps of both clades: mean shapes of host associated groups (landmarks and lines) are compared with the mean shape of the entire clade (deformation grid).

**FIGURE 4:**
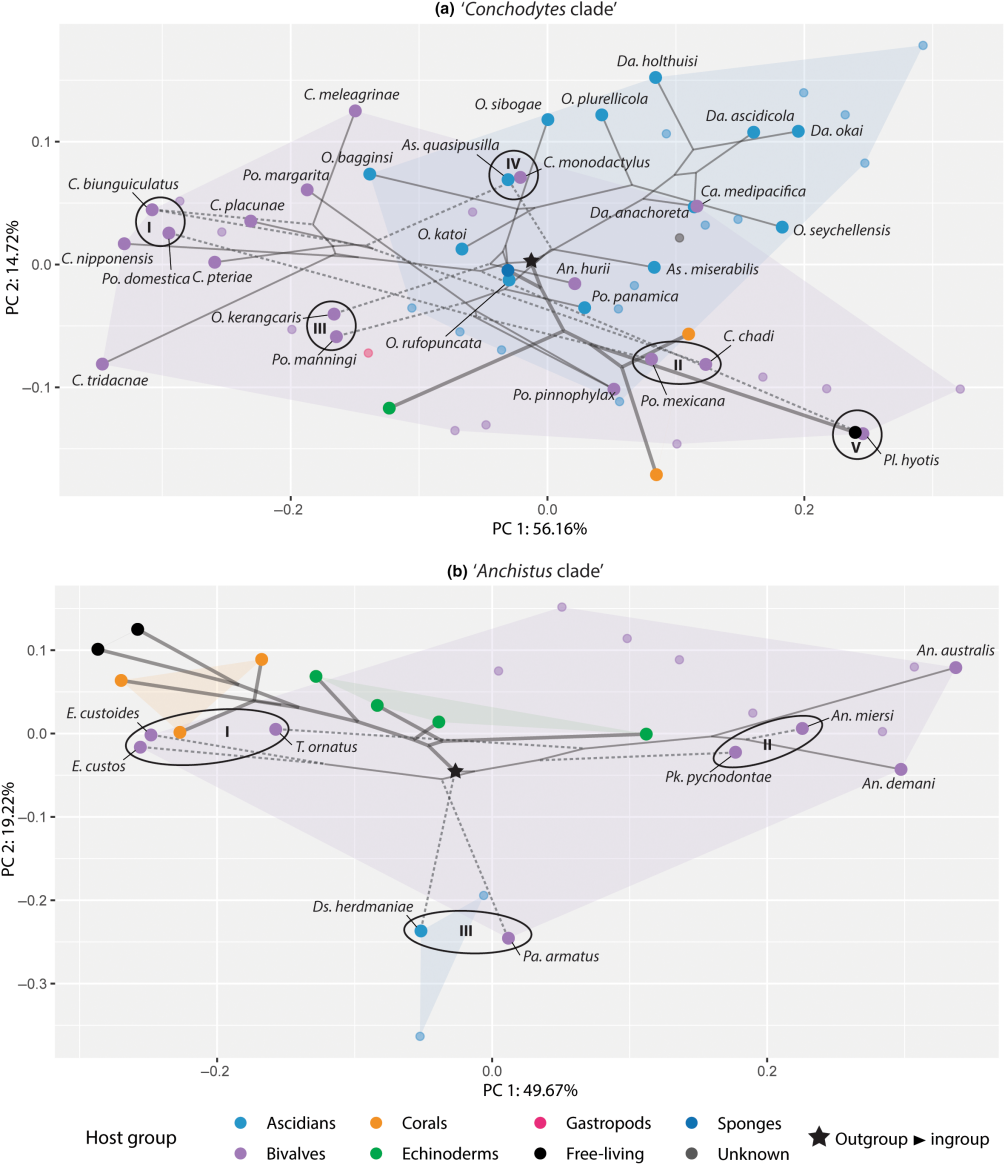
Phylomorphospaces of the third pereiopod dactylus shape variation, for both studied clades. (A) ‘*Conchodytes* clade’ (including five outgoups), (B) ‘*Anchistus* clade’ (including nine outgroups). Trimmed phylogeny trees from de Gier et al. (2022) and de Gier and Fransen (2023) are projected on the morphospaces; large datapoints are included in the phylogeny reconstructions, smaller datapoints make up the convex hulls of Figure 2. Colours indicate host association, and the black star indicate the split between in‐ (thin lines) and outgroups (wide lines). Possible convergence events are indicated with roman numerals, and the concerned species are encircled. Terminal branches of the (possibly) converging species are indicated with dotted lines. Genera names are abbreviated: (A) *An*., *Anchiopontonia*; *As*., *Ascidonia*; *C*., *Conchodytes*; Ca., *Cainonia*; *Da*., *Dactylonia*; *O*., *Odontonia*; *Pl*., *Platypontonia*; *Po*., *Pontonia*, (B) *An*., *Anchistus*; *Ds*., *Dasella*; *E*., *Ensiger*; *Pa*., *Paranchistus*; *Pk*., *Polkamenes*; *T*., *Tympanicheles*.

Procrustes (M)ANOVA tests were performed (in all PC dimensions) to test for the effect of host association on the placement of the species in the morphospaces. A significant effect was found for both clades (‘*Conchodytes* clade’: *R* ^2^ = .248, *p* = .001; ‘*Anchistus* clade’: *R* ^2^ = .411, *p* = .001), thus the host associations seem to be influencing the shape variation. The pairwise tests resulted in significant shape differences between the bivalve‐ and ascidian‐associated species, as well for both the clades (Figure 4): ‘*Conchodytes* clade’: *d* = 0.177, UCL (95%) = 0.097, Pr > *d* = 0.001; ‘*Anchistus* clade’: *d* = 0.337, UCL (95%) = 0.273, Pr > *d* = 0.013). As was mentioned above, the number of ascidian‐associated species in the ‘*Anchistus* clade’ is relatively low and the power of these statistical tests is therefore limited. Visual inspection, however, indicates a strong difference in the dactylus morphology of these species compared with the bivalve‐associated species in the clade (Figures 2b and 3). These morphological differences can be found in the shape of the unguis and the distance between the accessory tooth and the ventral unguis border. For the ‘*Conchodytes* clade’, the differences are less conspicuous: only the shape of the corpus influences the deformation grid to expand in height (Figure 3).

### 3.2 Phylomorphospaces

Several suspected convergence events have been selected and annotated in the phylomorphospace plots (roman numerals; I–V, and I–III, Figure 4). Stayton's similarity‐based measures for convergence (C_1_ to C_4_, and a *p*‐value for C_1_) for these species pairs can be found below (Table 1).

**TABLE 1:** Similarity‐based measures of convergence for eight presumed convergence events in the two studied clades (1000 replicates, PC1 to PC3; 81% and 80% of the data explained, for both clades, respectively).

Species combinations	C_1_	C_2_	C_3_	C_4_	*p*‐Value for C_1_
‘*Conchodytes* clade’					
(I) *Conchodytes biunguiculatus* (I/II)^a^ & *Pontonia domestica*	0.703/0.733	0.215/0.250	0.272/0.281	0.046/0.053	.019*/.014*
(II) *Conchodytes chadi* & *Pontonia mexicana*	0.765	0.254	0.335	0.054	.011*
(III) *Odontonia kerangcaris* & *Pontonia manningi*	0.813	0.219	0.354	0.047	.002*
(IV) *Ascidonia quasipusilla* & *Conchodytes monodactylus*	0.563	0.115	0.202	0.025	.037*
(V) *Cuapetes tenuipes* ^b^ & *Platypontonia hyotis*	0.828	0.243	0.539	0.044	.003*
‘*Anchistus* clade’					
(I) *Ensiger custoides* & *Tympanicheles ornatus*	0.751	0.312	0.376	0.190	.003*
(II) *Anchistus miersi* & *Polkamenes pycnodontae*	0.648	0.237	0.330	0.313	.016*
(III) *Dasella herdmaniae* & *Paranchistus armatus*	0.698	0.195	0.452	0.105	.010*

*Note*: *p*‐values under .05 are considered significant and are indicated with an asterisk (*).

^a^ Two values are given for both the species combinations with *Conchodytes biunguiculatus* (Paulson, 1875) I and II.

^b^ *Cuapetes tenuipes* (Borradaile, 1898) is part of the outgroup.

In the ‘*Conchodytes* clade’, the outgroups are placed in the bottom and right part of the plot, but the ingroup clade, and therefore the phylogeny projection, ‘starts’ in the middle of the plot (Figure 4A). The tree splits into two clades: one consisting of *Ascidonia*, *Dactylonia* and *Pontonia*, and the other of *Conchodytes* and *Odontonia* (also including one species each of *Anchiopontonia* Bruce, 1992 and *Platypontonia* Bruce, 1968) (Figure 4A). Within this first clade, the ascidian‐associated *Dactylonia* and *Ascidonia* cluster together, diverging within the ascidian‐associated convex hull. *Pontonia* also diverges within this convex hull, first with the ascidian associate *Pontonia panamica* Marin & Anker, 2008. Later, the branches split off in both directions, with *P*. *mexicana* and *P*. *pinnophylax* diverging towards a more elongated dactylus shape, and *Pontonia domestica* Gibbes, 1850; *Pontonia manningi* Fransen, 2000; and *Pontonia margarita* Smith in Verrill, 1869 towards a rounder dactylus shape (Figure 2).

The other major branch splits near the starting point of the phylogeny in the morphospace, into two clades: one including *Conchodytes* and basally *Platypontonia*, and the other *Odontonia* with basally *Anchiopontonia*. *Odontonia* and *Anchiopontonia* cluster somewhat together in the centre and top right part of the morphospace, with species of *Odontonia* making up the outer edges of the ascidian‐associated convex hull. The only exception to this is the bivalve‐associated *Odontonia kerangcaris* Fransen et al., 2021, diverging towards various species of *Conchodytes* and *Pontonia*. The branch including *Conchodytes* and *Platypontonia* splits near the middle of the morphospace, towards the right side of the plot with *Platypontonia* (with an elongated dactylus), and towards the left side with the species of *Conchodytes*. The divergence of *Conchodytes* from there resembles *Pontonia* (see above), with *Conchodytes biunguiculatus* (Paulson, 1875), *Conchodytes nipponensis* (De Haan, 1844), *Conchodytes placunae* (D.S. Johnson, 1967), *Conchodytes pteriae* Fransen, 1994 and *Conchodytes tridacnae* Peters, 1852 clustering together in the left side of the plot, and *Conchodytes chadi* (Marin, 2011) and, to some extent, *Conchodytes monodactylus* Holthuis, 1952, diverging to the right side.

In the phylomorphospace plot of the ‘*Anchistus* clade’, the phylomorphospace is less tangled (Figure 4B). After the somewhat clustered outgroup, the first clade within the ingroup including *Dasella* splits off towards the lower side of the plot. Here, *Dasella* is only represented by one species, *Dasella herdmaniae* (Lebour, 1938). After *Dasella*, the tree splits into two: one branch including both species of *Ensiger* and *Paranchistus armatus*, and one branch including *Anchistus*, *Polkamenes* and *Tympanicheles* (of which the latter two are only represented by one species). *Ensiger* diverges from the centre of the plot towards the left side, near the cnidarian‐associated outgroups, indicating that they have an elongated dactylus, without accessory tooth. *Paranchistus armatus* clusters together with *Dasella*, due to the large ventral outgrowth of the corpus of this species. The other major branch first splits off into *Polkamenes pycnodontae*, of which the dactylus resembles the more derived species of the ingroup, such as *Anchistus miersi* (De Man, 1888). Afterwards, *Tympanicheles ornatus* splits of in the direction of *Ensiger* and the cnidarian‐associated outgroups. The rest of the species of the branch, *A*. *miersi*, *Anchistus australis* Bruce, 1977, and *A*. *demani*, cluster together on the right side of the plot, characterized by a broad unguis.

The PGLS analyses resulted in insignificant values for both the ‘*Conchodytes* clade’ (*p* = .749; *R* ^2^ = .102) and the ‘*Anchistus* clade’ (*p* = .346; *R* ^2^ = .263). This means that the landmark (shape) data and thus the placement of the selected species within the morphospace is not associated with a host group, once phylogenetic non‐independence is taken into account. The *p*‐values of the regular ANOVA regressions came back as significant for both clades (‘*Conchodytes* clade’: *p* = .001; *R* ^2^ = .312; ‘*Anchistus* clade’: *p* = .024; *R* ^2^ = .402). This gives the impression that, for this subset of the original data, the phylogenetic framework is influencing the separation of our landmark (shape) data, with respect to host choice.

### 3.3 Dactylar microstructures

**FIGURE 5:**
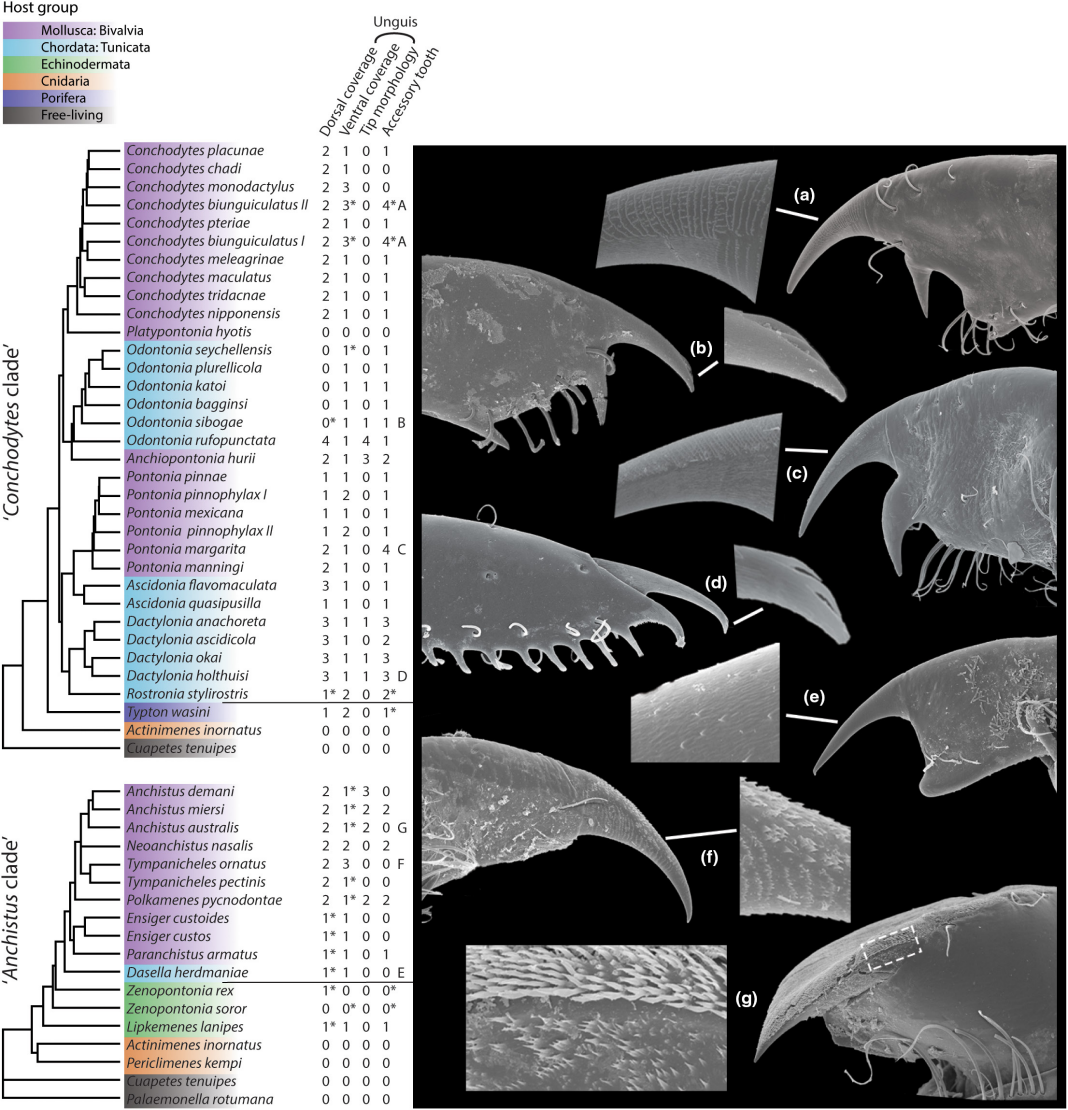
Character states of microstructures on the third pereiopod dactyli of the species in the studied clades, with SEM‐captures of examples of microstructure morphologies. Trimmed TE phylogeny trees from de Gier et al. (2022) and de Gier and Fransen (2023) are used. Four character states are scored, for the character states, and asterisks were added for character states which were tentatively placed in that category (see Section 2). Letters a–g correspond to the SEM‐captures on the right: see also Figures S5–S11. SEM‐captures not to scale. Colours indicate host association of the species, with *Zenopontonia rex* also being associated with nudibranch gastropods. The dactylus of *Odontonia sibogae* (Bruce, 1973) (b) after de Gier and Fransen (2018).

The hidden diversity of microstructures on the third pereiopod dactyli of the studied species was presented by the SEM‐captures (Figure 5 and Figures S5–S11). The dorsal coverage of the unguis could range from no microstructures in most outgroups, as well as in most species of *Odontonia*. In two outgroup species, sparsely placed minute teeth could be seen, which was a character shared by the genera *Dasella* (Figure 5e; on the proximal dorsal surface of the unguis), *Paranchistus*, *Ensiger*, but also in the other clade, in *Rostronia* Fransen, 2002. More, but irregular, coverage could be seen in most species of *Pontonia*, and in *Ascidonia quasipusilla* (Chace, 1972). A conspicuous patterning in the dorsal coverage was found not only in most species of *Conchodytes* (Figure 5a), but also in *Anchiopontonia hurii*, *Pontonia manningi* and *P*. *margarita* (Figure 5c). A comparable coverage was found in the rest of the species in the ‘*Anchistus* clade’, covering the entire dorsal surface of the unguis in the included species of *Anchistus* (Figure 5g) and *Polkamenes*.

The ventral coverage of the unguis follows a similar pattern in the phylogeny as the dorsal coverage: no coverage was found in most outgroups, while a dense coverage of shallow grooves was found in most ingroup species (Figures S5–S11). Randomly placed teeth were found in *Pontonia pinnophylax* and *Neoanchistus nasalis* Holthuis, 1986 (Figures S7e and S10c). In addition, patterning of the teeth on the ventral side of the unguis was found in *Tympanicheles ornatus* (Figure 5f) and *Conchodytes biunguiculatus* (Figure 5a). In the latter, the patterning stops halfway the unguis (Figure 5a).

The tip of the unguis can not only be pointed, as in most studied species (Figures S5–S11), but also be flattened and shaped like a scoop‐like structure. This character state was found in *Anchiopontonia hurii*, as well as in *Anchistus demani* (Figures S6a and S10f). The unguis is in these cases fully covered with minute teeth. A low number of large ‘scales’ on the unguis tip was found in species of *Odontonia*, such as *Odontonia sibogae* (Bruce, 1973) (Figure 5b), and in various species of *Dactylonia*, such as *Dactylonia holthuisi* Fransen, 2002 (Figure 5f).

The accessory tooth also shows some variation in microstructure coverage. A morphotype with an accessory tooth covered with shallow grooves and teeth on the proximal base was found: three species of *Dactylonia* share this character state (see *D*. *holthuisi*, Figure 5d). An accessory tooth covered with minute teeth was found in *Anchiopontonia hurii* and *Dactylonia ascidicola* (Borradaile, 1898), and in the other clade in *Anchistus miersi*, *Neoanchistus nasalis* and *Polkamenes pycnodontae* (Figures S9e and S10c,e). The accessory tooth of *Rostronia stylirostris* (Holthuis, 1952) shows a coverage with large teeth, a unique feature (Figure S8a). Both the species *Conchodytes biunguiculatus* and *Pontonia margarita* show a patterning of minute teeth only on the distal side of the accessory tooth, placed in patches (Figures S5f and S7d).

## 4 DISCUSSION AND CONCLUSIONS

### 4.1 Convergence in dactylus shapes and host switching in a morphospace

Although the pairwise Procrustes (M)ANOVA resulted in significant values when comparing the mean shapes of the bivalve‐ and ascidian‐associated species in both clades, there still seems to be an overlap in convex hulls of the first two PCs (Figure 2). One of these overlaps is also translated in the mean shapes to be almost the same in the ‘*Conchodytes* clade’, with the bivalve‐associated species having a somewhat more inflated corpus (Figure 3). The question arises if there are features in the walking leg dactyli that are typical for a certain host association, and how much of the variation is explained by the phylogenetic placement of the species. In the ‘*Conchodytes* clade’, both the ascidian‐ and bivalve‐associated groups feature species with a large accessory tooth (most species of *Conchodytes* and *Pontonia panamica*), a proportionally elongated unguis (most species of *Conchodytes*, *Pontonia* and *Pseudopontonia minuta* (Baker, 1907)), or a very flattened, elongated corpus (e.g. *Conchodytes chadi* and *Odontonia seychellensis* Fransen, 2002) (Figure 2a). The outgroup species do not cluster together in these first two PCs, and are scattered within the point clouds of both major host‐associated groups (Figure 2a). In the ‘*Anchistus* clade’, the same variation from an elongated dactylus shape (e.g. in *Ensiger*) to a more robust, inflated shape (e.g. in *Anchistus* and *Polkamenes*) can be found in the more numerous bivalve‐associated species (Figure 2b). Less variation can be found in the ascidian‐associated species: *Dasella* shows low intrageneric variation for the three known species (Figure 2b). In the ‘*Anchistus* clade’, the mean shapes for the host‐associated groups are easier to distinguish (Figure 3), and the outgroup species group somewhat together, although the overlap with the bivalve‐associated species point cloud is evident in the echinoderm‐associated species (Figure 2b).

The variation in shapes of both the ascidian‐ and bivalve‐associated species, as well as the placement of the outgroups in the presented morphospaces, gives us the impression that, in general, there is no evidence for a typical ‘bivalve‐ or ascidian‐associated morphotype’. This was already suggested by a previous study from Chow et al. (2021), wherein character mapping was performed for a whole range of symbiotic shrimp dactylus shapes, grouped into nine categories (Chow et al., 2021). Most bivalve‐ and ascidian‐associated species studied here (within clade IIIC and IIIH, sensu Chow et al., 2021) were grouped into category I (having no or a reduced accessory tooth) or II (the typical ‘biunguiculate’ dactylus shape), which they shared with numerous coral‐, echinoderm‐ and sponge‐associated species. In addition, a lot of free‐living species also share these general morphotypes. Four other species from the ‘*Conchodytes* clade’ were found in the monotypic category IV for the bivalve‐associated *Anchiopontonia hurii* (having a scoop‐like unguis), and in category V for the species with a serrated ventral corpus. This was the case for the bivalve‐associated *Cainonia medipacifica*, and the ascidian‐associated *Dactylonia ascidicola* and *Odontonia sibogae*. This last category was shared with numerous sponge‐, and echinoderm‐associated species. *Dasella herdmaniae* was placed together with the included species of the coral‐associated *Jocaste* Holthuis, 1952 and *Coralliocaris* Stimpson, 1860 in category VIII, due to the distoventral dilatation of the corpus. As can be seen, the morphotypes of the included bivalve‐ and ascidian‐associated species are shared by a wide range of symbionts from related, and unrelated, clades. However, if the similarities between these morphologically similar species and our studied ingroup species are homologous, and if they share the same functional morphology, they need to be studied in more detail. It is also worth noting that other endosymbiotic shrimp lineages show a lot of intra‐generic variation in dactylus shape (as can be seen in the related, species‐rich, and ascidian‐ and sponge‐endosymbiotic genera *Periclimenaeus* Borradaile, 1915 and *Typton* Costa, 1844; see Bruce, 2000, 2013: fig. 5E & 8H; Neves, 2020: fig. 3C; Anker et al., 2021: fig. 8). In the analysis by Chow et al. (2021), the morphological variation in these genera seems to be conserved, possibly due to the sampling size (as is the case of our ingroup species).

When following the phylogeny projections on our phylomorphospace plots (Figure 4), it can be deduced that there have been major morphological radiations in the evolution of the studied branches, even with the limited number of included species. Several potential instances of convergent evolution have been highlighted and statistically tested (Figure 4, Table 1). These convergences, and the link with the host‐switching events described in previous studies (de Gier et al., 2022; de Gier & Fransen, 2023), are discussed below.

Starting with the ‘*Conchodytes* clade’ (Figure 4A), the genera *Conchodytes*, *Odontonia* and *Pontonia* seem to be spreading out the most in the phylomorphospace. Various convergence events between species of *Conchodytes* and *Pontonia* can be discerned: *P*. *domestica* and *P*. *margarita* occur on the ‘typical’ *Conchodytes*‐side of the plot, at PC1_min_ (Figure 4A: event I); and *C*. *chadi*, *P*. *mexicana* and *P*. *pinnophylax* all share a more elongated dactylus, on the positive side of PC1 (Figure 4A: event II). Moreover, *P*. *manningi* can also be found on the negative side of PC1, together with the only bivalve‐associated *Odontonia* species, *O*. *kerangcaris* (see Figure 4A: event III). Closer examination of the dactylus of *O*. *kerangcaris* (see Fransen et al., 2021) shows a very ‘*Conchodytes*‐like’ dactylus for this species. The aberrant host association and the basal placement of the bivalve‐associated *O*. *kerangcaris* might be the cause of its placement close to various species of *Conchodytes* and *Pontonia*, instead of closer to its ascidian‐associated congeners. The convergence between the two groups of *Conchodytes* and *Pontonia* cannot be explained by their host choice; they do not share the same host genera. Possible convergence between two species from different genera, and with different host associations could be found between *Ascidonia quasipusilla* and *Conchodytes monodactylus* (Figure 4A: event IV). No apparent reason was found for this very apparent‐looking convergence event; the host of *C*. *monodactylus* is shared with more species within the genus (from *Pinna* and *Pteria*). The shapes of the dactyli do not match at first sight, it seems the shape of the unguis placed *C*. *monodactylus* between the ascidian‐associated species in the first two principal components. When plotting the second and third principal component, the datapoints are placed more separately (Figure S3). The last convergence event in this plot can be observed to have happened between *Platypontonia hyotis* Hipeau‐Jacquotte, 1971, and the included free‐living outgroup species *Cuapetes tenuipes* (Borradaile, 1898) (Figure 4A: event V). This indicates an evolutionary pathway from a more complex dactylus shape, to a simple, elongated shape. This has not only happened in this first clade, but also twice in the ‘*Anchistus* clade’, with both species of *Ensiger* and *Tympanicheles ornatus* moving towards the direction of the cnidarian‐associated and free‐living outgroups (PC1_min_) (Figure 4B: event I). It is worth noting that *T*. *ornatus* does, however, show more complex microstructures than the other species with an elongated dactylus. Another convergence event was found between the seemingly complex dactylus shapes of *Anchistus miersi* (and the other species of *Anchistus*) and *Polkamenes pycnodontae*, towards the right side of the plot (PC1_max_) (Figure 4B: event II). In addition, two different genera with different host associations (*Dasella* and *Paranchistus*) have evolved similar dactylus shapes, according to the plot (Figure 4B: event III). This might be the result of the basal placement of the species, which will be discussed below.

When mapping the host‐switching events from previous studies (de Gier et al., 2022; de Gier & Fransen, 2023) on the (limited) phylomorphospace data, it can be noted that the switches from an ascidian to a bivalve association result in morphological divergence in these clades. In other words, after host‐switches, bivalve‐associated species appear to be edging towards the limits of PC1, thereby increasing the morphological disparity. This is the case for the initial switch from an ascidian‐associated ancestor for the branch including *Conchodytes*, *Odontonia* and related genera, towards a bivalve association in *Conchodytes*, *Anchiopontonia hurii* and *O*. *kerangcaris* (de Gier et al., 2022: Figure 4, event V). After this initial switch, the branch including the other *Odontonia* species remains somewhat conservative in its morphological disparity, limited to the upper‐right section of the phylomorphospace (Figure 4A). The same happens in the branch including *Ascidonia*, *Dactylonia* and *Pontonia*, which branches out in the top right and middle part of the phylomorphospace, until the switch from ascidian association towards bivalve association takes place after *P*. *panamica* (Figure 4A). The other *Pontonia* species, similar to *Conchodytes*, spread out towards the morphological limits of PC1. Within the ‘*Anchistus* clade’, this cannot be seen due to the ancestral state being recovered as bivalve‐associated (de Gier & Fransen, 2023). However, the bivalve‐associated species have diversified much after the initial switch from ectosymbiosis towards endosymbiosis, covering a wide range of the morphospace (Figure 4B). Host‐switches have been proven to be driving force behind diversification in symbiotic shrimp (Chow et al., 2021; Horká et al., 2016), which can now also be quantified on a morphological scale in our phylomorphospace plots.

### 4.2 Evolution and diversification of microstructures

The character matrix of the microstructure morphotypes shows some evolutionary patterning of microstructures on the walking leg dactyli of the included species (Figure 5). Evidence for convergence in microstructure coverage was found within the clades. First up, convergence towards a complex, regular patterning of scales or teeth on the dorsal and ventral side of the unguis was found in various bivalve‐associated lineages (Figure 5a,c,f,g). Most striking is the convergence between various species of *Conchodytes* (e.g. *C*. *biunguiculatus*; Figure 5a) and *Pontonia* (*P*. *margarita*; Figure 5c and *P*. *manningi*; Figure S7C). This convergence of microstructures among these species was also seen in the overall dactylus shape (Figure 5a, also for *P*. *domestica*). No similarity between the host taxa of the species with this particular microstructure morphotype was found, including shrimp species of which no SEM‐images were taken, but are known to have similar microstructures (e.g. *Pontonia panamica*; *Pontonia simplex* Holthuis, 1951; and *Pontonia pilosa* Fransen, 2002 (Fransen, 2002; Marin & Anker, 2008)). This morphotype, although in this case possibly not homologous, was also found in the more derived branches of the ‘*Anchistus* clade’. In *Tympanicheles* and *Neoanchistus*, these scales still somewhat resemble the ones in *Conchodytes* and *Pontonia*, while in *Anchistus* and *Polkamenes*, the scales formed a regular coverage on the full dorsal surface of the unguis, accompanied by the lateral expansion of the unguis and corpus (Figures S5, S7, S9, S10). This second type of coverage was already discussed and compared by Fujino (1975) and Bruce (1980), accompanied by SEM‐captures.

The selected outgroup species might give an indication of the evolutionary pathway the species in our clades have undergone to show such elaborate microstructures. The free‐living and cnidarian‐associated species appear to have no microstructures on their unguis, while the sponge symbiont *Typton wasini* Bruce, 1977 (Figure S11a) displays very fine scales on the dorsal and ventral base. Both echinoderm‐associated *Lipkemenes lanipes* (Kemp, 1922) and *Zenopontonia rex* (also associated with nudibranches) exhibit very disperse scales on the dorsal side of the unguis, a character shared with some basal species of the ‘*Anchistus* clade’ (*Dasella herdmaniae*, both species of *Ensiger* and *Paranchistus armatus*). This is in contrast with the unguis morphology of the other two species of *Zenopontonia*: *Z*. *soror* (Nobili, 1904) (Figure S11c) and *Z*. *noverca* (Kemp, 1922) (pers. obs.), which have no dorsal microstructures. *Z*. *soror* only has proximally placed microstructures on the ventral side of the unguis (Figure S11c); *Zenopontonia* already appears to be polyphyletic in published phylogeny reconstructions (Chow et al., 2021; Horká et al., 2016), for which the SEM‐photographs might warrant taxonomic reappraisal. The presence of microstructures in somewhat related outgroups already shows that these features are not unique to our ingroup species. Moreover, inspection of SEM images from dactyli of species in the unrelated, also endosymbiotic, clade of *Periclimenaeus* shows elaborate microstructure patterning in the ascidian‐associated *Periclimenaeus storchi* Bruce, 1989, but not the sponge‐associated *Periclimenaeus bidentatus* Bruce, 1970 (pers. obs.). Whether the elaborate microstructures are strictly found in endosymbiotic lineages of the palaemonid family tree remains unclear.

Five ascidian‐associated species from different lineages within the ‘*Conchodytes* clade’ exhibit the same morphotype for the unguis tip (Figure 5b,d). This convergence towards an unguis tip with only a small number of large scales has happened at least once in both *Odontonia* and *Dactylonia* (de Gier & Fransen, 2018; Figures S6 and S8). This morphotype could tentatively be linked to the host choice on a lower taxonomic level. The species *Ascidonia quasipusilla* and all species of *Odontonia* (excluding the bivalve‐associated *O*. *kerangcaris* and phlebobranch‐associate *Odontonia plurellicola* de Gier & Fransen, 2018) reside inside of ascidians from the order Stolidobranchia, and appear to have no or limited microstructures on the surface of their unguis (de Gier & Fransen, 2018, see also Figure 5). This character state is also shared with the stolidobranch‐associated genus *Dasella* (Figure 5e). In contrast, three species within *Dactylonia* and *Ascidonia flavomaculata* (Heller, 1864) can be found in ascidians from the order Phlebobranchia. These species share an intricate pattern of rows on the dorsal surface of the unguis (Figures S7a and S8). This feature is, however, shared with *Dactylonia anachoreta* (Kemp, 1922) (Figure S8e), which lives in stolidobranch ascidians. In addition, the phlebobranch‐associated *O*. *plurellicola* (Figure S6g) does not share this morphotype. Excluding these two exceptions, it might be proposed that the internal anatomical differences between the two ascidian orders might influence the selection pressures for the symbionts (e.g. for easy movement in the host).

Similar to the overall dactylus shape evolution discussed above, plotting the host‐switching events on the tree might help to understand when microstructure diversification has occurred. In the ‘*Conchodytes* clade’, the variation in microstructures appears to be more apparent in the bivalve‐associated species, with the exception of *Platypontonia* showing no complex structures whatsoever (Figure S5a). Similar to the evolution of the dactylus shapes, a host‐switching event at the base of the clade containing *Conchodytes*, *Odontonia* and the smaller related genera appears to have jumpstarted a wide range of microstructure differences within *Conchodytes* (and *Anchiopontonia*). After this initial switch towards a bivalve host, the clade of *Odontonia* switched back to an ascidian host, and with it losing most microstructures. This switch seems to have happened somewhere between *Anchiopontonia* and *O*. *rufopunctata*, with the latter having an ‘intermediate’ microstructure morphology (Figure S6b). *Odontonia kerangcaris* (which is basal to *Odontonia rufopunctata* Fransen, 2002) also shows regularly placed teeth on the dorsal surface of the unguis (Fransen et al., 2021). Moreover, the switch between an ascidian host towards a bivalve host in the branch containing *Ascidonia*, *Dactylonia* and *Pontonia* seems to have caused some diversification of microstructures in *Pontonia*. Hypothetically, this switch should have happened in the branch after the ascidian‐associated *P*. *panamica*, but the original description of the species shows regular microstructures at the dorsal base of the unguis (Marin & Anker, 2008). New observations using a light microscope prove this (pers. obs. on the paratypes: RMNH.CRUS.D.51824). This means that the evolution of complex microstructures possibly has happened before *P*. *panamica* splits off in the phylogeny reconstruction, possibly proven by *Ascidonia quasipusilla* having basic, but conspicuously placed teeth on the dorsal unguis surface (Figure S7b). As mentioned above, the first bivalve‐associated branch in the ‘*Anchistus* clade’, including *Ensiger* and *Paranchistus*, shows no elaborate microstructures. Theories about host switching cannot prove why this clade did not evolve similar microstructures as in the other clade, including *Anchistus*, *Neoanchistus*, *Polkamenes* and *Tympanicheles*. It is worth noting that the members of *Ensiger* and *Paranchistus* are usually larger in size than their relatives from the other genera, which might play a role in the grip within the host.

### 4.3 Functional morphology and its implications

The huge variation in dactylus shapes and microstructures is thought to be the result of different selection pressures in the different host groups (Chow et al., 2021). Although no true ‘bivalve‐ or ascidian‐specific morphotype’ could be identified, the shape of the dactyli might serve similar purposes in both endo‐ as well as ectosymbionts. For instance, the typical ‘*Conchodytes*‐like’, biunguiculate, dactylus shape is shared between various bivalve associates (e.g. in *Conchodytes* and *Pontonia*, but also in *Odontonia kerangcaris*; Figures S5b–h,j, S7c,d; Fransen et al., 2021), but also in various species of the ascidian‐ and sponge‐associated *Periclimenaeus*, like *Periclimenaeus djiboutensis* Bruce, 1970; *Periclimenaeus matherae* Bruce, 2005; and *Periclimenaeus pachydentatus* Bruce, 1969 (Bruce, 2013; Marin, 2007). As shown by Chow et al. (2021), dactyli with a large accessory tooth are also present in various ectosymbiotic clades. It is safe to assume that the accessory tooth is used to grasp host tissue for better grip where strong currents are at play. Interestingly, the convergence towards ‘simpler’ elongated dactylus shapes seen in the studied ingroups (e.g. in *Conchodytes chadi*, *Ensiger*, *Platypontonia*, *Odontonia seychellensis* and various species of *Pontonia*; Figures S5a,i, S6f, S7e,f, S9c,d) can also be observed in *Periclimenaeus* (see Bruce, 2013) and various other clades (Chow et al., 2021). The same applies for dactyli of which the ventral side is serrated, as can be seen in *Dactylonia*, *Odontonia* and *Rostronia* (Figures S6b–g, S8a–e; for comparison with *Periclimenaeus*, see Bruce, 2013). The microstructures might also play a role in the grip within the host specimens: it seems like endosymbiotic species (also including the third endosymbiotic lineage; sensu Chow et al., 2021) display more microstructures on the accessory teeth and unguis than their ectosymbiotic relatives (Figure S11; for *Periclimenaeus*, see Bruce, 2013). While the hosts are from completely different phyla and do not resemble each other externally, the mucus‐covered body walls of the pharyngeal basket of ascidians and the gill structures of bivalves do appear similar in structure and in function. This microhabitat is in both cases possibly selecting for symbionts with dactyli covered in elaborate micro‐ornamentations. This does however not explain the lack of elaborate microstructures in *Ensiger*, *Paranchistus* (Figure S9b–d) and various other species in our ingroups. A study focusing on the selection pressures within these microhabitats, comparing not only bivalves and ascidians but also other hosts for endosymbionts (in sponges, see above), might give insights to why some types of microstructures have evolved in these clades of symbionts.

The morphology of the other appendages may reflect the host choice of the symbionts as well. Chow et al. (2021) demonstrated that the variation mandible shape, although expected, did not show any link with host choice, or the type of symbiosis (endo‐ versus ectosymbiotic). The methods of food transport, different dietary compositions and weaker selection pressures might have influenced the mandibular diversity of the studied clades of palaemonid shrimp. The type of symbiosis might also be reflected by the dietary habits, and perhaps the morphology of the mouthparts (Ashelby et al., 2015; De Grave et al., 2021). For comparison, Chow et al. (2023) showed some links between the host choice and the mouthpart morphology of a large group of pea crabs (Pinnotheroidea), which were either filter‐feeding, or feeding from the host tissue and/or mucus. In contrast to the study by Chow et al. (2021), Dobson et al. (2014, 2016) suggest endosymbionts to have smaller and simpler eyes, compared to their free‐living and ectosymbiotic relatives. It is thought that this is the result of the protective symbiosis, where species do not need good eyesight to survive (Dobson et al., 2014). A study should be done to investigate the effect of the mating system (e.g. if the males or even females leave their host to seek for new mates) on the eye morphology of the currently studied species. For comparison, males of host‐hopping pea crab species have relatively larger eyes than the females (de Gier & Becker, 2020).

### 4.4 Limitations and future perspectives

The present study describes the variation in walking leg dactylus shape and unguis microstructure in two clades of palaemonid shrimp species. While the supposed convergence events uncovered in this study are studied extensively, it is inevitable that major patterns were missed due to a lack of known morphological and ecological data. First, incomplete sampling of some of the rare species resulted in a limited dataset. For example, sometimes only one specimen of a species was caught (e.g. *Conchodytes philippinensis* Bruce, 1996; see Bruce, 1996), or no host records are known (*Pontonia longispina*; see Fransen, 2002). In addition, some species are also thought to be synonyms of well‐known species (*Polkamenes spondylis* (Suzuki, 1971) and *Polkamenes nobilii* (Holthuis, 1952) possibly being variations of *Polkamenes pycnodontae*; see de Gier & Fransen, 2023) or are ‘cryptic species’ and part of so‐called species complexes, in which multiple species might be waiting to be discovered (polyphyletic species in the phylogeny reconstruction in de Gier et al., 2022). This all leads to noise in our usable dataset, causing us to exclude species from the analyses, and consequently to miss possible instances of host‐switching and general convergence patterning in morphological structures.

Incomplete sampling also causes us to have insufficient molecular data for phylogeny reconstructions. This was, however, bypassed by implementing TE analyses in de Gier et al. (2022) and de Gier and Fransen (2023) to build phylogenetic trees using molecular and morphological information, which were used here in the phylomorphospace analyses. Species of which only morphological data were available were excluded from the analyses in the phylomorphospace. Although ‘trimming’ the tree disables us to calculate convergence events with these excluded species, some hypotheses can be drawn using the dactylar (phylo)morphospaces (Figures 2 and 4 and Figures S2 and S3) and TE topologies from de Gier et al. (2022) and de Gier and Fransen (2023). For instance, in the ‘*Conchodytes* clade’, we highlighted only two convergence events towards a simple elongated dactylus shape (Figure 4A: convergence events II and V), but this can also be seen in at least two other lineages within the clade (excluding *Platypontonia brevirostris* (Miers, 1884) which possibly is closely related to *P*. *hyotis*). Two other *Pontonia* species (*P*. *simplex* and *P*. *pilosa*), and to some extent, *Opaepupu huna* Anker & De Grave, 2021, all exhibit elongated dactyli similar to the ones seen in the free‐living outgroups (see Anker & De Grave, 2021; Fransen, 2002), indicating two to three new possible convergence events. In the ‘*Anchistus* clade’, similar events can also be observed in *Neoanchistus nasalis*, grouping together with *Anchistus australis*, and in *Neoanchistus cardiodytes* Bruce, 1975, which groups together in the top middle part of the plot with two species of *Polkamenes*, and *Anchistus gravieri* Kemp, 1922. These placements also indicate possible convergence events between three genera within the clade.

More sampling efforts might help us in building better and naturally sound phylogeny reconstructions, placing rarely caught species in existing trees (van der Meij et al., 2023). As can be seen in this study, using these phylogeny reconstructions in combination with morphological data, gathered through historical as well as recent museum collections, helps us in understanding when and how convergent evolution of adaptive features occurred in symbiotic clades. Palaemonid shrimps have already been suggested to be preferred model organisms to study mating systems and speciation under sympatric conditions (Baeza et al., 2015; Chow et al., 2021), but this study proves that this speciose and morphologically variable clade can also be used to study convergent evolution on a small scale. In addition, this knowledge should be supplemented with studies on the microhabitats of the symbionts, as they might give us insights in why symbionts have evolved in such intricate ways. The same can be mentioned for other symbiotic invertebrate groups, like certain clades of parasitic molluscs that live in association with corals, also occupying specific places in or on their hosts (Gittenberger & Gittenberger, 2005, 2011; Gittenberger & Hoeksema, 2013; Owada & Hoeksema, 2011; Potkamp et al., 2017).

## AUTHOR CONTRIBUTIONS

**Werner de Gier:** Conceptualization (equal); data curation (equal); formal analysis (equal); funding acquisition (equal); investigation (equal); methodology (lead); resources (equal); software (equal); supervision (equal); validation (equal); visualization (equal); writing – original draft (lead); writing – review and editing (lead). **Pepijn Helleman:** Data curation (equal); investigation (equal); resources (equal); visualization (equal); writing – review and editing (equal). **Jurriaan van den Oever:** Data curation (equal); investigation (equal); resources (equal); visualization (equal); writing – review and editing (equal). **Charles H. J. M. Fransen:** Conceptualization (equal); project administration (lead); resources (equal); supervision (equal); validation (equal); writing – review and editing (equal).

## CONFLICT OF INTEREST STATEMENT

All authors have no competing interests to declare.

## Supporting information


Appendix S1.

## Data Availability

The data that support the findings of this study are available in the Supporting Information of this article.

## References

[ece310768-bib-0001] Adams, D. , Collyer, M. L. , Kaliontzopoulou, A. , & Baken, E. (2022). Geomorph: Software for geometric morphometric analyses. R package version 4.0.4.

[ece310768-bib-0002] Adams, D. C. , & Collyer, M. L. (2018). Multivariate comparative methods: Evaluations, comparisons, and recommendations. Systematic Biology, 67, 14–31. 28633306 10.1093/sysbio/syx055

[ece310768-bib-0003] Anker, A. , & De Grave, S. (2021). *Opaepupu*, a new genus and species of bivalve‐associated shrimp (Decapoda: Caridea: Palaemonidae) from Hawai'i. Zootaxa, 4903(1), 55–70. 10.11646/zootaxa.4903.1.3 33757105

[ece310768-bib-0004] Anker, A. , Pachelle, P. P. G. , & Leray, M. (2021). Two new species of *Typton* Costa, 1844 from tropical American waters, with taxonomic notes on *T*. *tortugae* McClendon, 1911 and a new record of *T*. *granulosus* Ayón‐Parente, Hendrickx & Galvan‐Villa, 2015 (Decapoda: Caridea: Palaemonidae). Zootaxa, 4950(2), 267–295. 10.11646/zootaxa.4950.2.3 33903438

[ece310768-bib-0005] Ashelby, C. W. , De Grave, S. , & Johnson, M. L. (2015). Preliminary observations on the mandibles of palaemonoid shrimp (Crustacea: Decapoda: Caridea: Palaemonoidea). PeerJ, 3, e846. 10.7717/peerj.846 25825676 PMC4375974

[ece310768-bib-0006] Baeza, J. A. (2015). Crustaceans as symbionts: An overview of their diversity, host use and life styles. In L. Watling & M. Thiel (Eds.), The life styles and feeding biology of the Crustacea (pp. 163–189). Oxford University Press.

[ece310768-bib-0007] Baeza, J. A. , Hemphill, C. A. , & Ritson‐Williams, R. (2015). The sexual and mating system of the shrimp *Odontonia katoi* (Palaemonidae, Pontoniinae), a symbiotic guest of the ascidian *Polycarpa aurata* in the Coral Triangle. PLoS One, 10(3), e0121120. 10.1371/journal.pone.0121120 25799577 PMC4370848

[ece310768-bib-0008] Baken, E. , Collyer, M. L. , Kaliontzopoulou, A. , & Adams, D. (2021). Geomorph v4.0 and gmShiny: Enhanced analytics and a new graphical interface for a comprehensive morphometric experience. Methods in Ecology and Evolution, 12, 2355–2363. 10.1111/2041-210X.13723

[ece310768-bib-0009] Bruce, A. J. (1972). Shrimps that live with molluscs. Sea Frontiers, 18, 218–227.

[ece310768-bib-0010] Bruce, A. J. (1975). Notes on some Indo‐Pacific Pontoniinae. XXVI. *Neoanchistus cardiodytes* gen. nov. sp. nov., a new mollusc‐associated shrimp from Madagascar (Decapoda: Palaemonidae). Crustaceana, 29, 149–165.

[ece310768-bib-0011] Bruce, A. J. (1976). Coral Reef Caridea and ‘Commensalism’. Micronesica, 12, 83–98.

[ece310768-bib-0012] Bruce, A. J. (1980). SEM observations on the ambulatory dactyls of some pontoniine shrimps (Decapoda Caridea). Crustaceana, 38(2), 178–182.

[ece310768-bib-0013] Bruce, A. J. (1983). Additions to the marine fauna of the Northern Territory. 1. Decapod Crustacea: Caridea and Stenopodidaea. The Beagle, Occasional Papers of the Northern Territory Museum of Arts and Sciences, 1(5), 41–49.

[ece310768-bib-0014] Bruce, A. J. (1990). Crustacea Decapoda: Deep‐sea Palaemonoid shrimps from New Caledonian waters. In: Crosnier, A. (Ed.) Résultats des' Campagnes MUSORSTOM, Volume 6. Mémoires du Muséum National d'Histoire Naturelle. Série A. Zoologie, 145, 149–215. Paris, France.

[ece310768-bib-0015] Bruce, A. J. (1992). Designation of two new pontoniine shrimp genera (Decapoda: Palaemonidae). Journal of Natural History, 26(6), 1273–1282. 10.1080/00222939200770721

[ece310768-bib-0016] Bruce, A. J. (1995). A synopsis of the Indo‐West Pacific genera of the Pontoniinae (Crustacea, Decapoda, Palaemonidae). Koeltz Scientific Books.

[ece310768-bib-0017] Bruce, A. J. (1996). Crustacea Decapoda: Palaemonoid shrimps from the Indo‐Pacific region mainly from New Caledonia. In: Crosnier, A. (ed.) Résultats des Campagnes MUSORSTOM, volume 15. Mémoires du Muséum National d'Histoire naturelle. Série A, Zoologie, 168, 197–267.

[ece310768-bib-0018] Bruce, A. J. (2000). *Typton manningi* and *T*. *capricorniae*, new species, new pontoniine shrimps from northern Queensland, with a review of the Indo‐West Pacific species of *Typton* Costa (Decapoda: Palaemonidae). Journal of Crustacean Biology, 20, special number 2, 87–100.

[ece310768-bib-0019] Bruce, A. J. (2013). Identification aid for the Indo‐West Pacific species of *Periclimenaeus* Borradaile, 1915 (Crustacea: Decapoda: Caridea: Pontoniinae) using ambulatory dactyli. Memoirs of the Queensland Museum, 56, 647–664.

[ece310768-bib-0020] Burin, G. , Kissling, W. , Guimarães, P. R. , Şekercioğlu, Ç. H. , & Quental, T. B. (2016). Omnivory in birds is a macroevolutionary sink. Nature Communications, 7, 11250. 10.1038/ncomms11250 PMC4829659 27052750

[ece310768-bib-0021] Chow, L. H. , Ahyong, S. T. , Lam, Y. F. , Naruse, T. , Ng, P. K. L. , & Tsang, L. M. (2023). Shift in symbiotic lifestyle as the major process shaping the evolution of pea crabs (Decapoda: Brachyura: Pinnotheroidea). Molecular Phylogenetics and Evolution, 188, 107904. 10.1016/j.ympev.2023.107904 37579893

[ece310768-bib-0022] Chow, L. H. , De Grave, S. , & Tsang, L. M. (2021). Evolution of protective symbiosis in palaemonid shrimps (Decapoda: Caridea) with emphases on host spectrum and morphological adaptations. Molecular Phylogenetics and Evolution, 162, 107201. 33984469 10.1016/j.ympev.2021.107201

[ece310768-bib-0023] Collyer, M. L. , & Adams, D. C. (2021). RRPP: Linear Model Evaluation with Randomized Residuals in a Permutation Procedure, R package version 1.1.2.

[ece310768-bib-0024] Davis, K. E. , De Grave, S. , Delmer, C. , & Wills, M. A. (2018). Freshwater transitions and symbioses shaped the evolution and extant diversity of caridean shrimps. Communications Biology, 1, 16. 10.1038/s42003-018-0018-6 30271903 PMC6123698

[ece310768-bib-0025] de Gier, W. (2023). Phylomorphometrics reveal ecomorphological convergence in pea crab carapace shapes (Brachyura, Pinnotheridae). Ecology and Evolution, 13, e9744. 10.1002/ece3.9744 36694551 PMC9842789

[ece310768-bib-0026] de Gier, W. , & Becker, C. (2020). A review of the ecomorphology of pinnotherine pea crabs (Brachyura: Pinnotheridae), with an updated list of symbiont‐host associations. Diversity, 12(11), 431. 10.3390/d12110431

[ece310768-bib-0027] de Gier, W. , & Fransen, C. H. J. M. (2018). *Odontonia plurellicola* sp. n. and *Odontonia bagginsi* sp. n., two new ascidian‐associated shrimp from Ternate and Tidore, Indonesia, with a phylogenetic reconstruction of the genus (Crustacea, Decapoda, Palaemonidae). ZooKeys, 765, 123–160. 10.3897/zookeys.765.25277 PMC6002420 29910665

[ece310768-bib-0028] de Gier, W. , & Fransen, C. H. J. M. (2023). Polka‐dotted treasures: Revising a clade of ascidian‐ and bivalve‐associated shrimps (Caridea: Palaemonidae). Contributions to Zoology, 92(3), 1–104.

[ece310768-bib-0029] de Gier, W. , Groenhof, M. , & Fransen, C. H. J. M. (2022). Coming out of your shell or crawling back in: Multiple interphylum host switching events within a clade of bivalve‐ and ascidian‐associated shrimps (Caridea: Palaemonidae). Contributions to Zoology, 91(3), 166–198. 10.1163/18759866-bja10030

[ece310768-bib-0030] De Grave, S. (2001). Biogeography of Indo‐Pacific Pontoniinae (Crustacea, Decapoda): A PAE analysis. Journal of Biogeography, 28(10), 1239–1253.

[ece310768-bib-0031] De Grave, S. , Struck, U. , & Johnson, M. L. (2021). Preliminary study into the trophic position of symbiotic palaemonid shrimps (Decapoda, Palaemonidae) using stable isotopes. Crustaceana, 94, 1145–1153.

[ece310768-bib-0032] Dobson, N. C. , De Grave, S. , & Johnson, M. L. (2014). Linking eye design with host symbiont relationships in pontoniine shrimps (Crustacea, Decapoda, Palaemonidae). PLoS One, 9, e99505. 24950292 10.1371/journal.pone.0099505 PMC4064969

[ece310768-bib-0033] Dobson, N. C. , Johnson, M. L. , & De Grave, S. (2016). Insights into the morphology of symbiotic shrimp eyes (Crustacea, Decapoda, Palaemonidae); the effects of habitat demands. PeerJ, 4, e1926. 27168962 10.7717/peerj.1926 PMC4860300

[ece310768-bib-0034] Doña, J. , Proctor, H. , Mironov, S. , Serrano, D. , & Jovani, R. (2018). Host specificity, infrequent major host switching and the diversification of highly host‐specific symbionts: The case of vane‐dwelling feather mites. Global Ecology and Biogeography, 27, 188–198. 10.1111/geb.12680

[ece310768-bib-0035] Edelaar, P. , Jovani, R. , & Gomez‐Mestre, I. (2017). Should I change or should I go? Phenotypic plasticity and matching habitat choice in the adaptation to environmental heterogeneity. The American Naturalist, 190(4), 506–520. 10.1086/693345 28937819

[ece310768-bib-0036] Fransen, C. H. J. M. (1994). Shrimps and Molluscs./Garnalen en Weekdieren. Vita Marina, 42(4), 105–113.

[ece310768-bib-0037] Fransen, C. H. J. M. (2002). Taxonomy, phylogeny, historical biogeography, and historical ecology of the genus *Pontonia* (Crustacea: Decapoda: Caridea: Palaemonidae). Zoologische Verhandelingen, 336, 1–433.

[ece310768-bib-0038] Fransen, C. H. J. M. , Groenhof, M. , & de Gier, W. (2021). *Odontonia kerangcaris* sp. nov., a new bivalve‐associated shrimp (Crustacea, Decapoda, Palaemonidae) from East Kalimantan, revealing intrageneric host switching. Zootaxa, 5081(2), 275–285. 35391009 10.11646/zootaxa.5081.2.6

[ece310768-bib-0039] Fransen, C. H. J. M. , & Reijnen, B. T. (2013). Caught in speciation? A new host for *Conchodytes meleagrinae* Peters, 1852 (Decapoda, Caridea, Palaemonidae). Zootaxa, 3721(3), 265–280. 26120672 10.11646/zootaxa.3721.3.3

[ece310768-bib-0040] Fransen, C. H. J. M. , van der Veer, E. , & Frolová, P. (2022). A new species of scleractinian associated shrimp of the genus *Palaemonella* (Crustacea, Decapoda, Palaemonidae) with a redescription of *Palaemonella orientalis* Dana, 1852. Zootaxa, 5214(4), 557–580. 37044890 10.11646/zootaxa.5214.4.5

[ece310768-bib-0041] Frolová, P. , Horká, I. , & Ďuriš, Z. (2022). Molecular phylogeny and historical biogeography of marine palaemonid shrimps (Palaemonidae: Palaemonella‐Cuapetes group). Scientific Reports, 12, 15237. 10.1038/s41598-022-19372-5 36075944 PMC9458662

[ece310768-bib-0042] Fujino, T. (1975). Fine features of the dactylus of the ambulatory pereiopods in a bivalve associated shrimp, *Anchistus miersi* (De Man), under the scanning electron microscope (Decapoda Natantia, Pontoniinae). Crustaceana, 29(3), 252–254.

[ece310768-bib-0043] Gan, Z. B. , Li, X. Z. , Chan, T.‐Y. , Chu, K. H. , & Kou, Q. (2015). Phylogeny of Indo‐West Pacific pontoniine shrimps (Crustacea: Decapoda: Caridea) based on multilocus analysis. Journal of Zoological Systematics and Evolutionary Research, 53, 282–290.

[ece310768-bib-0044] Ghalambor, C. K. , Mckay, J. K. , Carroll, S. P. , & Reznick, D. N. (2007). Adaptive versus non‐adaptive phenotypic plasticity and the potential for contemporary adaptation in new environments. Functional Ecology, 21, 394–407. 10.1111/j.1365-2435.2007.01283.x

[ece310768-bib-0045] Gittenberger, A. , & Gittenberger, E. (2005). A hitherto unnoticed adaptive radiation: Epitoniid species (Gastropoda: Epitoniidae) associated with corals (Scleractinia). Contributions to Zoology, 74(1–2), 125–203. 10.1163/18759866-0740102009

[ece310768-bib-0046] Gittenberger, A. , & Gittenberger, E. (2011). Cryptic, adaptive radiation of endoparasitic snails: Sibling species of *Leptoconchus* (Gastropoda: Coralliophilidae) in corals. Organisms Diversity & Evolution, 11(1), 21–41. 10.1007/s13127-011-0039-1

[ece310768-bib-0047] Gittenberger, A. , & Hoeksema, B. W. (2013). Habitat preferences of coral‐associated wentletrap snails (Gastropoda: Epitoniidae). Contributions to Zoology, 82(1), 1–25. 10.1163/18759866-08201001

[ece310768-bib-0048] Gómez‐Robles, A. , Olejniczak, A. J. , Martinón‐Torres, M. , Prado‐Simón, L. , & Bermúdez de Castro, J. M. (2011). Evolutionary novelties and losses in geometric morphometrics: A practical approach through hominin molar morphology. Evolution, 65(6), 1772–1790. 10.1111/j.1558-5646.2011.01244.x 21644962

[ece310768-bib-0049] Goto, R. , Kawakita, A. , Ishikawa, H. , Hamamura, Y. , & Kato, M. (2012). Molecular phylogeny of the bivalve superfamily Galeommatoidea (Heterodonta, Veneroida) reveals dynamic evolution of symbiotic lifestyle and interphylum host switching. BMC Evolutionary Biology, 12, 172. 10.1186/1471-2148-12-172 22954375 PMC3532221

[ece310768-bib-0050] Gotto, V. (2004). Commensal and Parasitic Copepods associated with Marine Invertebrates: Keys and Notes for the Identification of British Species. In J. H. Crothers & P. J. Hayward (Eds.), Synopses of the British Fauna (New Series), 46 (2nd ed.). Field Studies Council.

[ece310768-bib-0051] Hayashi, K.‐I. (2003). Prawns, shrimps and lobsters from Japan (131). Family Palaemonidae, subfamily Pontoniinae – Genera *Hamodactyloides* and *Pontonia* . Aquabiology, 148, 25(5), 366–369. (In Japanese).

[ece310768-bib-0052] Horká, I. , De Grave, S. , Fransen, C. H. J. M. , Petrusek, A. , & Ďuriš, Z. (2016). Multiple host switching events shape the evolution of symbiotic palaemonid shrimps (Crustacea: Decapoda). Scientific Reports, 6, 26486. 10.1038/srep26486 27246395 PMC4887867

[ece310768-bib-0053] Horká, I. , De Grave, S. , Fransen, C. H. J. M. , Petrusek, A. , & Ďuriš, Z. (2018). Multiple origins and strong phenotypic convergence in fish‐cleaning palaemonid shrimp lineages. Molecular Phylogenetics and Evolution, 124, 71–81. 10.1016/j.ympev.2018.02.006 29501373

[ece310768-bib-0054] Jablonski, D. (2008). Species selection: Theory and data. Annual Review of Ecology, Evolution, and Systematics, 39, 501–524.

[ece310768-bib-0055] Joy, J. B. (2013). Symbiosis catalyses niche expansion and diversification. Proceedings of the Royal Society B, 280, 20122820. 10.1098/rspb.2012.2820 23390106 PMC3574373

[ece310768-bib-0056] Kise, H. , Alves Santos, M. E. , Fourreau, C. J. L. , Iguchi, A. , Goto, R. , & Reimer, J. D. (2023). Evolutionary patterns of host switching, lifestyle mode, and the diversification history in symbiotic zoantharians. Molecular Phylogenetics and Evolution, 182, 107732. 36781031 10.1016/j.ympev.2023.107732

[ece310768-bib-0057] Komai, T. , Tsuchida, S. , & Fujiwara, Y. (2023). A new deep‐sea palaemonid shrimp assigned to *Periclimenes* Costa, 1844 (Decapoda: Caridea) from the West Mariana Ridge, northwestern Pacific. Zootaxa, 5231(4), 376–392. 37045138 10.11646/zootaxa.5231.4.2

[ece310768-bib-0058] Kou, Q. , Li, X. Z. , Chan, T. Y. , & Chu, K. H. (2015). Divergent evolutionary pathways and host shifts among the commensal pontoniine shrimps: A preliminary analysis based on selected Indo‐Pacific species. Organisms Diversity & Evolution, 15, 369–377.

[ece310768-bib-0059] Kou, Q. , Li, X. Z. , Chan, T. Y. , Chu, K. H. , Huang, H. , & Gan, Z. (2013). Phylogenetic relationships among genera of the *Periclimenes* complex (Crustacea: Decapoda: Pontoniinae) based on mitochondrial and nuclear DNA. Molecular Phylogenetics and Evolution, 68(1), 14–22. 23535017 10.1016/j.ympev.2013.03.010

[ece310768-bib-0060] Li, H. , Sosa‐Calvo, J. , Horn, H. A. , Pupo, M. T. , Clardy, J. , Rabeling, C. , Schultz, T. R. , & Currie, C. R. (2018). Convergent evolution of complex structures for ant–bacterial defensive symbiosis in fungus‐farming ants. PNAS, 115(42), 10720–10725. 10.1073/pnas.1809332115 30282739 PMC6196509

[ece310768-bib-0061] Losos, J. , & Ricklefs, R. (2009). Adaptation and diversification on islands. Nature, 457, 830–836. 10.1038/nature07893 19212401

[ece310768-bib-0062] Marin, I. (2007). Pontoniine shrimps (Decapoda: Caridea: Palaemonidae) inhabiting boring sponges (Porifera: Demospongia) from Nhatrang Bay, Vietnam, with description of three new species. Zoologische Mededelingen, 81(12), 217–240.

[ece310768-bib-0063] Marin, I. , & Anker, A. (2008). A new species of *Pontonia* Latreille, 1829 (Crustacea, Decapoda, Palaemonidae) associated with seasquirts (Tunicata, Ascidiacea) from the Pacific coast of Panama. Zoosystema, 30, 501–515.

[ece310768-bib-0064] Munday, P. L. , van Herwerden, L. , & Dudgeon, C. L. (2004). Evidence for sympatric speciation by host shift in the sea. Current Biology, 14(16), 1498–1504. 15324668 10.1016/j.cub.2004.08.029

[ece310768-bib-0065] Mundry, R. (2014). Statistical issues and assumptions of phylogenetic generalized least squares. In L. Z. Garamszegi (Ed.), Modern phylogenetic comparative methods and their application in evolutionary biology. Springer. 10.1007/978-3-662-43550-2_6

[ece310768-bib-0066] Neves, K. (2020). A new species of the shrimp genus *Typton* Costa, 1844 (Malacostraca, Decapoda, Palaemonidae) from the Cabo Verde archipelago. Zootaxa, 4768(2), 264–270. 10.11646/zootaxa.4768.2.7 33056527

[ece310768-bib-0067] Owada, M. , & Hoeksema, B. W. (2011). Molecular phylogeny and shell microstructure of *Fungiacava eilatensis* Goreau et al. 1968, boring into mushroom corals (Scleractinia: Fungiidae), in relation to other mussels (Bivalvia: Mytilidae). Contributions to Zoology, 80(3), 169–178. 10.1163/18759866-08003001

[ece310768-bib-0068] Pagel, M. (1999). Inferring the historical patterns of biological evolution. Nature, 401, 877–884. 10.1038/44766 10553904

[ece310768-bib-0069] Pérez‐Losada, M. , Høeg, J. T. , & Crandall, K. A. (2009). Remarkable convergent evolution in specialized parasitic Thecostraca (Crustacea). BMC Biology, 7, 15. 10.1186/1741-7007-7-15 19374762 PMC2678073

[ece310768-bib-0070] Potkamp, G. , Vermeij, M. J. , & Hoeksema, B. W. (2017). Genetic and morphological variation in corallivorous snails (*Coralliophila* spp.) living on different host corals at Curaçao, southern Caribbean. Contributions to Zoology, 86(2), 111–114. 10.1163/18759866-08602002

[ece310768-bib-0071] Poulin, R. , & Randhawa, H. S. (2015). Evolution of parasitism along convergent lines: From ecology to genomics. Parasitology, 142(Suppl 1), S6–S15. 10.1017/S0031182013001674 24229807 PMC4413784

[ece310768-bib-0072] R Core Team . (2022). R: A language and environment for statistical computing. R Foundation for Statistical Computing, Vienna, Austria. https://www.R‐project.org/

[ece310768-bib-0073] Rauch, C. , Hoeksema, B. W. , Hermanto, B. , & Fransen, C. H. J. M. (2019). Shrimps of the genus *Periclimenes* (Crustacea, Decapoda, Palaemonidae) associated with mushroom corals (Scleractinia, Fungiidae): Linking DNA barcodes to morphology. Contributions to Zoology, 88, 201–235. 10.1163/18759866-20191357

[ece310768-bib-0074] Rohlf, F. J. (2017). tpsDig2 v.2.27. State University of New York at Stony Brook.

[ece310768-bib-0075] RStudio Team . (2022). RStudio: Integrated Development for R. RStudio, PBC, Boston, MA., United States.

[ece310768-bib-0076] Sapp, J. (1994). Evolution by association: A history of Symbiosis (pp. 1–272). Oxford University Press.

[ece310768-bib-0077] Schluter, D. (2000). The ecology of adaptive radiation (pp. 1–296). Oxford University Press.

[ece310768-bib-0078] Stayton, C. T. (2015). The definition, recognition, and interpretation of convergent evolution, and two new measures for quantifying and assessing the significance of convergence. Evolution, 69, 2140–2153. 10.1111/evo.12729 26177938

[ece310768-bib-0079] Stayton, C. T. (2018). convevol: Analysis of convergent evolution. R package version 1.3.

[ece310768-bib-0080] van der Meij, S. E. T. , Bouwmeester, J. , & Bähr, S. (2023). DNA barcoding, dwelling morphology, and fecundity of the gall‐forming shrimp *Paratypton siebenrocki* Balss, 1914 (Caridea: Palaemonidae). Journal of Natural History, 57(1–4), 25–37. 10.1080/00222933.2022.2158383

[ece310768-bib-0081] Vehof, J. , van der Meij, S. E. T. , Tuerkay, M. , & Becker, C. (2016). Female reproductive morphology of coral‐inhabiting gall crabs (Crustacea: Decapoda: Brachyura: Cryptochiridae). Acta Zoologica, 97, 117–126.

[ece310768-bib-0082] Wickham, H. (2016). ggplot2: Elegant graphics for data analysis. Springer‐Verlag. 10.1007/978-0-387-98141-3_9

