# Supplementary material for: Ecomorphological convergence in the walking leg dactyli of two clades of ascidian‐ and mollusc‐associated shrimps (Decapoda: Caridea: Palaemonidae)
Source: Ecol Evol. 2023 Dec 19;13(12):e10768. doi: 10.1002/ece3.10768 (PMC10731117; doi:10.1002/ece3.10768)

Ecomorphological convergence in the walking leg dactyli of two clades of ascidian- and mollusc-associated shrimps (Decapoda: Caridea: Palaemonidae)

Werner de Gier^1,2^, Pepijn Helleman^1,3^, Jurriaan van den Oever^1,3^, Charles H. J. M. Fransen^1^

^1^: Naturalis Biodiversity Center, P.O. Box 9517, 2300 RA, Leiden, The Netherlands

^2^: Groningen Institute for Evolutionary Life Sciences, University of Groningen, P.O. Box 11103, 9700 CC Groningen, The Netherlands

^3^: Institute of Biology Leiden, Leiden University, P.O. Box 9505, 2300, RA Leiden, The Netherlands.

Supplementary material

Table S1 List of specimens and material used in the analyses of the current study.

List of specimens and material used in the analyses of the current study. Species and genera names, general host group, sources for the general dactylus shape illustrations, and collection registration numbers (Coll. nr.), specimen localities, and host associations are given for the SEM-study specimens. The column ‘Nr.’ links the species names to the datapoints in figure S1, for the ‘*Conchodytes* clade’ (Supplementary Figures 2a and 3a) and the ‘*Anchistus* clade’ (Supplementary Figures 2b and 3b); outgroups are given two numbers where needed, in the order as mentioned before (Supplementary Figure 2). Numbers between brackets are annotated to the species in the morphospace plot with both clades included (Supplementary Figure 4). Museum abbreviations: RMNH.CRUS.D. – Naturalis Biodiversity Center, former Rijksmuseum van Natuurlijke Historie, Leiden, The Netherlands; MZB – Museum Zoologicum Bogoriense, Bogor, Indonesia; MCZ – Museum for Comparative Zoology, Harvard, Cambridge, MA, USA. *) These sources are not published illustrations and/or sketches, and expedition reports of recent collection expeditions. *Dactylonia holthuisi* Fransen, 2002 is the only species for which the latter is used. Additional information for this specimen: Sta. BAL.23: Tulamben area, bay S of Emerald Hotel - 08°17’05’’S 115°36’11’’E; 30 m depth; 15.iv.2001; in *Plurella* sp. on sand; collected by C.H.J.M. Fransen. Material deposited in Naturalis Biodiversity Center*.* **) The dactylus illustration of *Periclimenes* *lacertae* Bruce, 1992, now *Cuapetes* *ischiospinosus* (Bruce, 1991), is used for *Cuapetes* *tenuipes* (Borradaile, 1898) due to their similar morphology.

| General information | | | Dactylus morphology | SEM study | | |
| --- | --- | --- | --- | --- | --- | --- |
| Species | **Nr.** | **Host group** | **Source** | **Coll. Nr.** | **Locality** | **Host record** |
| *CONCHODYTES CLADE* | | | | | | |
| *Anchiopontonia hurii* (Holthuis, 1981) | **6 (11)** | Bivalves | Bruce, 1992a | RMNH.CRUS.D.49842 | Talumben, Bali, Indonesia | *Spondylus versicolor* Schreibers, 1793 |
| *Ascidonia californiensis* (Rathbun, 1902) | **7 (12)** | Ascidians | Fransen, 2002 |  |  |  |
| *Ascidonia flavomaculata* (Heller, 1864) | **8 (13)** | Ascidians | Fransen, 2002 | RMNH.CRUS.D.45617 | Sal, Cape Verde Islands | Ascidiacea |
| *Ascidonia miserabilis* (Holthuis, 1951) | **9 (14)** | Ascidians | Fransen, 2002 |  |  |  |
| *Ascidonia pusilla* (Holthuis, 1951) | **10 (15)** | Ascidians | Fransen, 2002 |  |  |  |
| *Ascidonia quasipusilla* (Chace, 1972) | **11 (16)** | Ascidians | Fransen, 2002 | RMNH.CRUS.D.51538 | Stn. Al Awam, Mauritania | Ascidiacea |
| *Bruceonia ardeae* (Bruce, 1981) | **12 (17)** | Bivalves | Fransen, 2002 |  |  |  |
| *Cainonia medipacifica* (Edmondson, 1935) | **13 (18)** | Bivalves | Fransen, 2002 |  |  |  |
| *Colemonia litodactylus* Bruce, 2005 | **14 (19)** | Ascidians | Bruce, 2005 |  |  |  |
| *Conchodytes biunguiculatus* (Paulson, 1875) | **15+16 (20/21)** | Bivalves | Fransen, 1994 | RMNH.CRUS.D.53209 | Santo, Vanuatu | *Pinna atropurpurea* G.B. Sowerby I, 1825 |
| *Conchodytes chadi* (Marin, 2011) | **17 (22)** | Bivalves | Fransen & Reijnen, 2012 | RMNH.CRUS.D.53857 | Ligitan Island, Semporna, Sabah, Malaysia | *Lopha cristagalli* (L., 1758) |
| *Conchodytes kempoides* Bruce, 2013 | **18 (23)** | Bivalves | Bruce, 1989a |  |  |  |
| *Conchodytes maculatus* Bruce, 1989 | **19 (24)** | Bivalves | Bruce, 1989b | RMNH.CRUS.D.6526 | Jedan Island, Aru Islands, Indonesia | *Meleagrina* sp. |
| *Conchodytes meleagrinae* Peters, 1852 | **20+21 (25/26)** | Bivalves | Fransen & Reijnen, 2013 | RMNH.CRUS.D.57923 | Santo, Vanuatu | *Pinctada* sp. |
| *Conchodytes monodactylus* Holthuis, 1952 | **22 (27)** | Bivalves | Unpublished illustration* | RMNH.CRUS.D.53212 | Tikus Island, Kepulauan Seribu, Indonesia | *Pteria penguin* (Röding, 1798) |
| *Conchodytes nipponensis* (De Haan, 1844 [in De Haan, 1833-1850]) | **23 (28)** | Bivalves | Fransen, 1994 | RMNH.CRUS.D.57925 | Tikus Island, Kepulauan Seribu, Indonesia | *Atrina pectinidata* (L., 1767) |
| *Conchodytes philippinensis* Bruce, 1996 | **24 (29)** | Bivalves | Bruce, 1996 |  |  |  |
| *Conchodytes placunae* (D. S. Johnson, 1967) | **25 (30)** | Bivalves | Fransen, 1994 | RMNH.CRUS.D.53216 | Tikus Island, Kepulauan Seribu, Indonesia | *Placuna placenta* (L., 1758) |
| *Conchodytes pteriae* Fransen, 1994 | **26 (31)** | Bivalves | Fransen, 1994 | RMNH.CRUS.D.58033 | Talumben, Bali, Indonesia | *Pteria* sp. |
| *Conchodytes tridacnae* Peters, 1852 | **27 (32)** | Bivalves | Bruce, 1977a | RMNH.CRUS.D.42787 | St. Joseph atoll, Seychelles | *Tridacna* sp. |
| *Dactylonia anachoreta* (Kemp, 1922) | **28 (33)** | Ascidians | Fransen, 2002 | RMNH.CRUS.D.42556 | NW of Praslin Island, Seychelles | *Ascidia* sp. |
| *Dactylonia ascidicola* (Borradaile, 1898) | **29+30 (34/35)** | Ascidians | Fransen, 2002 | RMNH.CRUS.D.50551 | Berau Islands, NE Kalimantan, Indonesia | Ascidiacea |
| *Dactylonia borradalei* Bruce, 2005 | **31 (36)** | Ascidians | Bruce, 1996 |  |  |  |
| *Dactylonia carinicula* Bruce, 2006 | **32 (37)** | Ascidians | Bruce, 2006 |  |  |  |
| *Dactylonia franseni* Bruce, 2003 | **33 (38)** | Ascidians | Bruce, 2003a |  |  |  |
| *Dactylonia holthuisi* Fransen, 2002 | **34 (39)** | Ascidians | Fransen, 2002 | RMNH.CRUS.D.58031 | Selat Lembeh, N Sulawesi, Indonesia | *Plurella* sp. |
| *Dactylonia monnioti* (Bruce, 1990) | **35 (40)** | Ascidians | Fransen, 2002 |  |  |  |
| *Dactylonia okai* (Kemp, 1922) | **36 (41)** | Ascidians | Fransen, 2002 | RMNH.CRUS.D.48680 | Palau Serangan, Bali, Indonesia | Ascidiacea |
| *Notopontonia platycheles* Bruce, 1991 | **37 (42)** | Ascidians | Bruce, 1991 |  |  |  |
| *Odontonia bagginsi* de Gier & Fransen, 2018 | **38 (43)** | Ascidians | de Gier & Fransen, 2018 | MZB.Cru.4733 | Tidore, Halmahera, Indonesia | Ascidiacea |
| *Odontonia compacta* (Bruce, 1996) | **39 (44)** | Ascidians | Fransen, 2002 |  |  |  |
| *Odontonia katoi* (Kubo, 1940) | **40 (45)** | Ascidians | Fransen, 2002 | RMNH.CRUS.D.46701 | Spermonde Archipelago, SW Sulawesi, Indonesia | *Polycarpa aurata* (Quoy & Gaimard, 1834) |
| *Odontonia kerangcaris* Fransen, Groenhof & de Gier, 2021 | **41 (46)** | Bivalves | Fransen et al., 2021 |  |  |  |
| *Odontonia plurellicola* de Gier & Fransen, 2018 | **42 (47)** | Ascidians | de Gier & Fransen, 2018 | MZB.Cru.4734 | Ternate, Halmahera, Indonesia | *Plurella* sp. |
| *Odontonia rufopunctata* Fransen, 2002 | **43 (48)** | Ascidians | Fransen, 2002 | RMNH.CRUS.D.48694 | Spermonde Archipelago, SW Sulawesi, Indonesia | Ascidiacea |
| *Odontonia seychellensis* Fransen, 2002 | **44 (49)** | Ascidians | Fransen, 2002 | RMNH.CRUS.D.42762 | Mahe, Seychelles | Stolidobranchia |
| *Odontonia sibogae* (Bruce, 1973) | **45 (50)** | Ascidians | Fransen, 2002 | RMNH.CRUS.D.47581 | Ambon, Indonesia | *Polycarpa* sp. |
| *Odontonia simplicipes* (Bruce, 1996) | **46 (51)** | Ascidians | Fransen, 2002 |  |  |  |
| *Opaepupa huna* Anker & De Grave, 2021 | **47 (52)** | Bivalves | Anker & De Grave, 2021 |  |  |  |
| *Pinnotherotonia rumphiusi* Marin & Paulay, 2010 | **48 (53)** | Bivalves | Marin & Paulay, 2010 |  |  |  |
| *Platypontonia brevirostris* (Miers, 1884) | **49 (54)** | Bivalves | Bruce, 1968 |  |  |  |
| *Platypontonia hyotis* Hipeau-Jacquotte, 1971 | **50 (55)** | Bivalves | Hipeau-Jacquotte, 1971 | RMNH.CRUS.D.49848 | Talumben, Bali, Indonesia | *Hyotissa* sp. |
| *Pontonia chimaera* Holthuis, 1952 | **51 (56)** | Gastropods | Fransen, 2002 |  |  |  |
| *Pontonia domestica* Gibbes, 1850 | **52 (57)** | Bivalves | Fransen, 2002 |  |  |  |
| *Pontonia longispina* Holthuis, 1951 | **53 (58)** | Unknown | Fransen, 2002 |  |  |  |
| *Pontonia manningi* Fransen, 2000 | **54 (59)** | Bivalves | Fransen, 2002 | RMNH.CRUS.D.48667 | Terrefal, Sao Tiago, Cape Verde | *Spondylus senegalensis* Schreibers, 1793 |
| *Pontonia margarita* Smith in Verrill, 1869 | **55 (60)** | Bivalves | Fransen, 2002 | RMNH.CRUS.D.42581 | Golfo Duce, Costa Rica | *Pinctada mazatlantica* (Hanley, 1856) |
| *Pontonia mexicana* Guérin-Méneville, 1855 [in Guérin-Méneville, 1855-1856] | **56 (61)** | Bivalves | Fransen, 2002 | RMNH.CRUS.D.42578 | Frozen Key, Bahama Islands | *Pinna* sp. |
| *Pontonia panamica* Marin & Anker, 2008 | **57 (62)** | Ascidians | Marin & Anker, 2008 |  |  |  |
| *Pontonia pilosa* Fransen, 2002 | **58 (63)** | Bivalves | Fransen, 2002 |  |  |  |
| *Pontonia pinnae* Lockington, 1878 | **59 (64)** | Bivalves | Fransen, 2002 | MCZ.XXX | San Carlos Bay, Guaymas, Mexico | *Pinna rugosa* G.B. Sowerby I, 1835 |
| *Pontonia pinnophylax* (Otto, 1821) | **60+61 (65/66)** | Bivalves | Fransen, 2002 | RMNH.CRUS.D.42616 | Caldeira Inferno, Azores | Unknown host |
| *Pontonia simplex* Holthuis, 1951 | **62 (67)** | Bivalves | Fransen, 2002 |  |  |  |
| *Pseudopontonia minuta* (Baker, 1907) | **63 (68)** | Ascidians | Bruce, 2003b |  |  |  |
| *Rostronia stylirostris* (Holthuis, 1952) | **64 (69)** | Ascidians | Fransen, 2002 | RMNH.CRUS.D.42575 | Wadi Haart, Oman | Unknown host |
| *ANCHISTUS CLADE* |  |  |  |  |  |  |
| *Anchistus australis* Bruce, 1977 | **10 (70)** | Bivalves | De Gier & Fransen, 2023 | RMNH.CRUS.D.41287 | Teluk Dalam, Tioman Island, Malaysia | *Tridacna squamosa* Lamarck, 1819 |
| *Anchistus demani* Kemp, 1922 | **13 (73)** | Bivalves | De Gier & Fransen, 2023 | RMNH.CRUS.D.28584 | Marsa Murach, Egypt | Unknown host |
| *Anchistus gravieri* Kemp, 1922 | **14 (74)** | Bivalves | De Gier & Fransen, 2023 |  |  |  |
| *Anchistus miersi* (de Man, 1888 [in de Man, 1887-1888]) | **15 (75)** | Bivalves | De Gier & Fransen, 2023 | RMNH.CRUS.D.58030 | Kepulauan Seribu, Indonesia | *Tridacna squamosa* Lamarck, 1819 |
| *Dasella ansoni* Bruce, 1983 | **17 (77)** | Ascidians | Bruce, 1983 |  |  |  |
| *Dasella brucei* Berggen, 1990 | **18 (78)** | Ascidians | Bruce, 2003b |  |  |  |
| *Dasella herdmaniae* (Lebour, 1938) | **19 (79)** | Ascidians | De Gier & Fransen, 2023 | RMNH.CRUS.D.48251 | Spermonde Archipelago, SW Sulawesi, Indonesia | Ascidiacea |
| *Ensiger custoides* (Bruce, 1977) | **11 (71)** | Bivalves | De Gier & Fransen, 2023 | RMNH.CRUS.D.58029 | Selat Lembeh, N Sulawesi, Indonesia | *Atrina vexillum* (Born, 1778) |
| *Ensiger custos* (Forskål, 1775) | **12 (72)** | Bivalves | De Gier & Fransen, 2023 | RMNH.CRUS.D.57963 | Kepulauan Seribu, Indonesia | *Pinna bicolor* Gmelin, 1791 |
| *Neoanchistus cardiodytes* Bruce, 1975 | **20 (80)** | Bivalves | Bruce, 1975a |  |  |  |
| *Neoanchistus nasalis* Holthuis, 1986 | **21 (81)** | Bivalves | De Gier & Fransen, 2023 | RMNH.CRUS.D.48724 | Masicoh, Oman | Unknown host |
| *Paranchistus armatus* (H. Milne Edwards, 1837 [in Milne Edwards, 1834-1840]) | **22 (82)** | Bivalves | De Gier & Fransen, 2023 | RMNH.CRUS.D.46092 | Kei Islands, Tanimbar, Indonesia | *Tridacna gigas* (L., 1758) |
| *Polkamenes liui* (Li, Bruce & R.B. Manning, 2004) | **23 (83)** | Bivalves | Li et al., 2004 |  |  |  |
| *Polkamenes nobilii* (Holthuis, 1952) | **24 (84)** | Bivalves | De Gier & Fransen, 2023 |  |  |  |
| *Polkamenes pycnodontae* (Bruce, 1978) | **26 (86)** | Bivalves | De Gier & Fransen, 2023 | RMNH.CRUS.D.58032/ RMNH.CRUS.D.53787 | Kepulauan Seribu, Indonesia/Semporna, Sabah, Malaysia | *Hyotissa hyotis* (L., 1758)/*Spondylus* sp. |
| *Polkamenes spondylis* (Suzuki, 1971) | **27 (87)** | Bivalves | Suzuki, 1971 |  |  |  |
| *Tympanicheles ornatus* (Holthuis, 1952) | **25 (85)** | Bivalves | De Gier & Fransen, 2023 | RMNH.CRUS.D.41491 | Nocra Island, Eritrea | Unknown host |
| *Tympanicheles pectinis* (Kemp, 1925) | **16 (76)** | Bivalves | De Gier & Fransen, 2023 | RMNH.CRUS.D.47465 | Ambon, Indonesia | Pectinada |
| *OUTGROUPS* |  |  |  |  |  |  |
| *Actinimenes inornatus* (Kemp, 1922) | **3/9 (9)** | Cnidarians | Fransen, 1989 | RMNH.CRUS.D.42843 | St. Francois atoll, Seychelles | *Stichodactyla mertensii* Brandt, 1835 |
| *Actinimenes ornatus* (Bruce, 1969) | **4/8 (8)** | Cnidarians | Fransen, 1989 |  |  |  |
| *Cuapetes tenuipes* (Borradaile, 1898) | **1/1 (1)** | Free-living | Bruce, 1992b** | RMNH.CRUS.D.48784 | Bone Baku, Makassar, Sulawesi, Indonesia | (Free-living) |
| *Lipkemenes lanipes* (Kemp, 1922) | **-/5 (5)** | Echinoderms | Bruce & Okuno, 2010 | RMNH.CRUS.D.48452 | Cabilao Island, Cebu, Philippines | Euyalina |
| *Palaemonella rotumana* (Borradaile, 1898) | **-/2 (2)** | Free-living | De Gier & Fransen, 2023 | RMNH.CRUS.D.48378 | Cabilao Island, Cebu, Philippines | (Free-living) |
| *Periclimenes colemani* Bruce, 1975 | **2/6 (6)** | Echinoderms | Bruce, 1975b |  |  |  |
| *Periclimenes kempi* Bruce, 1969 | **-/7 (7)** | Cnidarians | Unpublished illustration* | RMNH.CRUS.D.47655 | Kotania bay, Seram, Indonesia | Ancylonacea |
| *Typton wasini* Bruce, 1977 | **5/- (10)** | Sponges | Bruce, 1977b | RMNH.CRUS.D.59346 | Selat Lembeh, N Sulawesi, Indonesia | *Callyspongia* sp. |
| *Zenopontonia rex* (Kemp, 1922) | **-/4 (4)** | Echinoderms/ Gastropods | Bruce, 1967 | RMNH.CRUS.D.42857 | St. Francois atoll, Seychelles | *Thelenota ananas* (Jaeger, 1833) |
| *Zenopontonia soror* (Nobili, 1904) | **-/3 (3)** | Echinoderms | Bruce, 1978 | RMNH.CRUS.D.59347 | Selat Lembeh, N Sulawesi, Indonesia | *Echinaster* sp. |

**References:**

Anker, A. & De Grave, S. (2021) *Opaepupu*, a new genus and species of bivalve-associated shrimp (Decapoda: Caridea: Palaemonidae) from Hawai'i. Zootaxa, 4903(1): 55-70.

Bruce, A. J. (1967) Notes on some Indo-Pacific Pontoniinae III-IX Descriptions of some new genera and species from the western Indian Ocean and the South China Sea ^1^). Zoologische Verhandelingen, 87: 1–73. [^1^) Contribution No. 12 from the Fisheries Research Station, Hong Kong.]

Bruce, A. J. (1968) Notes on some Indo-Pacific Pontoniinae. XII. The re-examination of the types of *Pontonia*? *brevirostris* Miers, 1884, with the designation of a new genus, *Platypontonia* (Decapoda, Natantia). Crustaceana, 15: 289–297.

Bruce, A. J. (1975a) Notes on some Indo-Pacific Pontoniinae. XXVI. *Neoanchistus cardiodytes* gen. nov., sp. nov., a new mollusk-associated shrimp from Madagascar (Decapoda, Palaemonidae). Crustaceana, 29(2): 149–165.

Bruce, A. J. (1975b) *Periclimenes colemani* sp. nov., a new shrimp associate of a rare sea urchin from Heron Island, Queensland (Decapoda Natantia, Pontoniinae). Records of the Australian Museum, 29(18): 486–501.

Bruce, A. J. (1977a) Pontoniine shrimps in the collections of the Australian Museum. Records of the Australian Museum, 31(2): 39–81.

Bruce, A. J. (1977b) Notes on some Indo-Pacific Pontoniinae, XXVIII. *Typton wasini* sp. nov., from Wasin Island, Kenya. Crustaceana, 32(3): 272–285.

Bruce, A. J. (1978) *Periclimenes soror* Nobili, a pontoniin shrimp new to the American Fauna, with observations on its Indo-West Pacific distribution. Tethys, 8(4): 299–306.

Bruce, A. J. (1983) A second species of the pontoniine shrimp genus *Dasella* Lebour, *D. ansoni* sp. nov., from the Arafura Sea. The Beagle, Occasional Papers of The Northern Territory Museum of Arts and Sciences, 1(3): 21–29.

Bruce, A. J. (1989a) Notes on some Indo-Pacfic Pontoniinae, XLV. *Conchodytes maculatus* sp. nov., a new bivalve associate from the Australian northwest shelf. Crustacea, 56(2): 182­–192.

Bruce, A. J. (1989b) A report on some coral reef shrimps from the Philippine islands. Asian Marine Biology, 6: 173–192.

Bruce, A. J. (1991) *Notopontonia platycheles*, new genus, new species (Decapoda: Pontoniinae) from South Australia, with remarks on *Pontonia pinnophylax* (Otto), the type species of *Pontonia* Latreille. Journal of Crustacean Biology, 11(4): 607–628.

Bruce, A. J. (1992a) Designation of two new pontoniine shrimp genera (Decapoda: Palaemonidae). Journal of Natural History, 26(6): 1273–1282. doi: 10.1080/00222939200770721

Bruce, A. J. (1992b) Two new species of *Periclimenes* (Crustacea: Decapoda: Palaemonidae) from Lizard Island, Queensland, with notes on some related taxa. Records of the Australian Museum, 44: 45–84.

Bruce, A. J. (1996) Crustacea Decapoda: Palaemonoid shrimps from the Indo-Pacific region mainly from New Caledonia. In: Crosnier, A. (Ed.) Résultats des Campagnes MUSORSTOM, Volume 15. Mémoires du Muséum national d'Histoire naturelle. Série A, Zoologie, 168: 197–267.

Bruce, A. J. (2003a) A new species of *Dactylonia* Fransen (Crustacea: Decapoda: Pontoniinae) from East Africa. Cahiers de Biologie Marine, 44: 299–306.

Bruce, A. J. (2003b) Further information on two pontoniine shrimps from ascidean hosts, *Dasella brucei* Berggren, 1990 and *Pseudopontonia minuta* (Baker, 1907) (Crustacea: Decapoda: Palaemonidae). Memoirs of the Queensland Museum, 49(1): 111–114.

Bruce, A. J. (2005) Pontoniine shrimps from Papua New Guinea, with designation of two new genera, *Cainonia* and *Colemonia* (Crustacea: Decapoda: Palaemonidae). Memoirs of the Queensland Museum, 51(2): 333–383.

Bruce, A. J. (2006) Pontoniine shrimps (Decapoda: Palaemonidae) from the island of Socotra, with descriptions of new species of *Dactylonia* Fransen, 2002 and *Periclimenoides* Bruce, 1990. Zootaxa, 1137: 1–36.

Bruce, A. J. & Okuno, J. (2010) Designation of a new genus *Lipkemenes*, with supplementary description and range extension of its type species, *L. lanipes* (Kemp, 1922) (Decapoda, Palaemonidae). In: Fransen, C. H. J. M., De Grave, S., Ng, P.K.L. (Eds.) Studies on Malacostraca: Lipke Bijdeley Holthuis Memory Volume. Brill, Leiden, The Netherlands. pp. 159–171.

de Gier, W. & Fransen, C. H. J. M. (2018) *Odontonia plurellicola* sp. n. and *Odontonia* *bagginsi* sp. n., two new ascidian-associated shrimp from Ternate and Tidore, Indonesia, with a phylogenetic reconstruction of the genus (Crustacea, Decapoda, Palaemonidae). ZooKeys, 765: 123–160.

de Gier, W. & Fransen, C. H. J. M. (2023) Polka-dotted treasures: revising a clade of ascidian- and bivalve-associated shrimps (Caridea: Palaemonidae). Contributions to Zoology, 92(3): 1–104.

Fransen, C. H. J. M. (1989) Notes on caridean shrimps collected during the Snellius-II Expedition. I. Associates of Anthozoa. Netherlands Journal of Sea Research, 23(2): 131–147.

Fransen, C. H. J. M. (1994) Marine Palaemonoid shrimps of the Netherlands Seychelles Expedition 1992-1993. Vol. 1. Zoologische Verhandelingen, 297: 85–152.

Fransen, C. H. J. M. (2002) Taxonomy, phylogeny, historical biogeography, and historical ecology of the genus *Pontonia* (Crustacea: Decapoda: Caridea: Palaemonidae). Zoologische Verhandelingen, 336: 1–433.

Fransen, C. H. J. M. & Reijnen, B. T. (2012) A second discovery of *Lacertopontonia chadi* Marin, 2011 (Crustacea: Decapoda: Palaemonidae), with remarks on its systematic position. Zootaxa, 3437: 43–50.

Fransen, C. H. J. M. & Reijnen, B. T. (2013) Caught in speciation? A new host for *Conchodytes* *meleagrinae* Peters, 1852 (Decapoda, Caridea, Palaemonidae). Zootaxa, 3721(3): 265–280.

Fransen, C. H. J. M., Groenhof, M., & de Gier, W. (2021) *Odontonia kerangcaris* sp. nov., a new bivalve-associated shrimp (Crustacea, Decapoda, Palaemonidae) from East Kalimantan, revealing intrageneric host switching. Zootaxa, 5081(2): 275–285.

Hipeau-Jacquotte, R. (1971) Notes de faunistique et de biologie marines de Madagascar, V. *Platypontonia hyotis* nov. sp. (Decapoda Natantia, Pontoniinae). Crustaceana 20(2): 125–140.

Li, X., Bruce, A. J., & Manning, R. B. (2004) Some palaemonid shrimps (Crustacea: Decapoda) from northern South China Sea, with descrip[i]tions of two new species. The Raffles Bulletin of Zoology, 52(2): 513–553.

Marin, I. & Anker, A. (2008) A new species of *Pontonia* Latreille, 1829 (Crustacea, Decapoda, Palaemonidae) associated with seasquirts (Tunicata, Ascidiacea) from the Pacific coast of Panama. Zoosystema, 30: 501–515.

Marin, I. & Paulay, G. (2010) *Pinnotherotonia rumphiusi* gen. et sp. nov., a new furry bivalve-associated pontoniine shrimp (Crustacea: Decapoda: Palaemonidae) from Palau. Zootaxa, 2636: 37–48.

Suzuki, H. (1971) On some commensal shrimps found in the western region of Sagami Bay. Researches on Crustacea, 4-5: 92–119.

Figure S1 List of landmarks for the morphometric analyses.

Landmark selection for the morphometric analyses on the third pair of pereiopod dactyli. Dactylus of *Pontonia manningi* Fransen, 2000 is shown with seven landmarks (filled in arrows) and 11 semi-landmarks (white dotted arrows). Drawing altered after Fransen (2002).


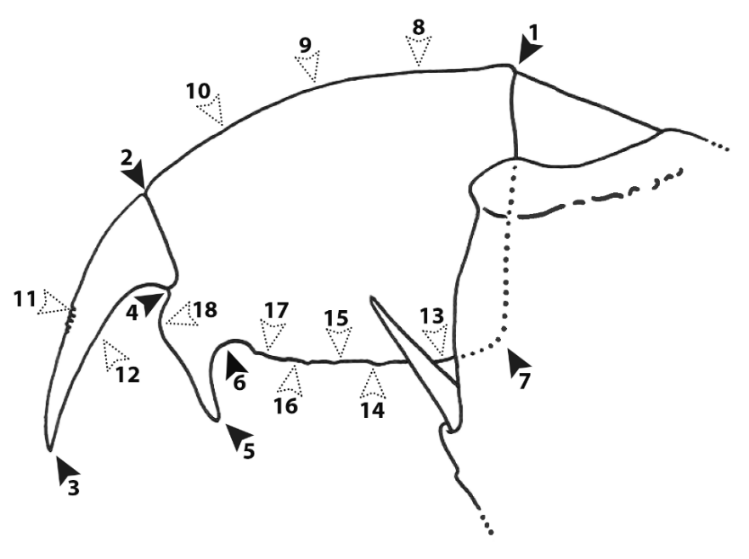


Landmarks:
1. The dorsoproximal end of the corpus, adjacent to the intersegmental membrane (approximation).
2. Dorsal border of the unguis with the corpus.
3. Terminal point of unguis.
4. Ventral border of the unguis with the corpus.
5. Terminal end of accessory tooth (in species without accessory tooth, same as landmark 4).
6. Proximal base of the accessory tooth (in species without accessory tooth, same as landmark 4).
7. The ventroproximal end of the corpus, adjacent to the intersegmental membrane
 (approximation).

Semi-landmarks:
8. Dorsal side of the corpus, quarter of the length to the unguis.
9. Dorsal side of the corpus, half of the length to the unguis.
10. Dorsal side of the corpus, three-quarter of the length to the unguis.
11. Dorsal side of the unguis, half of the length.
12. Ventral side of the unguis, half of the length.
13. Ventral side of the corpus, quarter of the length to the accessory tooth.
14. Ventral side of the corpus, half of the length to the accessory tooth.
15. Ventral side of the corpus, 5/8th of the length to the accessory tooth.
16. Ventral side of the corpus, three-quarter of the length to the accessory tooth.
17. Ventral side of the corpus, 7/8th of the length to the accessory tooth.
18. Distal base of accessory tooth (in species without accessory tooth, same as landmark 4).

List of used character states in SEM study.

- Unguis dorsal surface: coverage smooth (0); with randomly placed small teeth (1); with teeth placed in rows (2); with a striated pattern (3); and with a patch of teeth at the distal end only (4 – only for *Odontonia rufopunctata* Fransen, 2002).
- Unguis ventral surface: coverage smooth (0); with shallow grooves (1); with randomly placed small teeth (2); and with teeth placed in rows (3).
- Unguis tip morphology: simple (0); with one to three scale-like structures (1); with dorsal teeth also covering the unguis tip (2); with a scoop-like distal end, covered in teeth (3); and with a patch of teeth at the distal end only (4 – only for *O. rufopunctata*).
- Accessory tooth: absent, or barely visible (0); present, but without coverage (1); covered with minute teeth (2); covered with shallow grooves and teeth on the proximal base (3); with patches of minute teeth on the distal side (4).

Figure S2 Walking leg dactyli morphospace plots with datapoint numbers.

The plots from the morphospace analyses (1^st^ and 2^nd^ principal component), focussing on the walking leg dactyli morphology, are projected with datapoint numbers below. Numbers correspond with the numbers given in Table S1 (column: ‘Nr.’). Colours corresponds to host choices as in the figures in the main text.


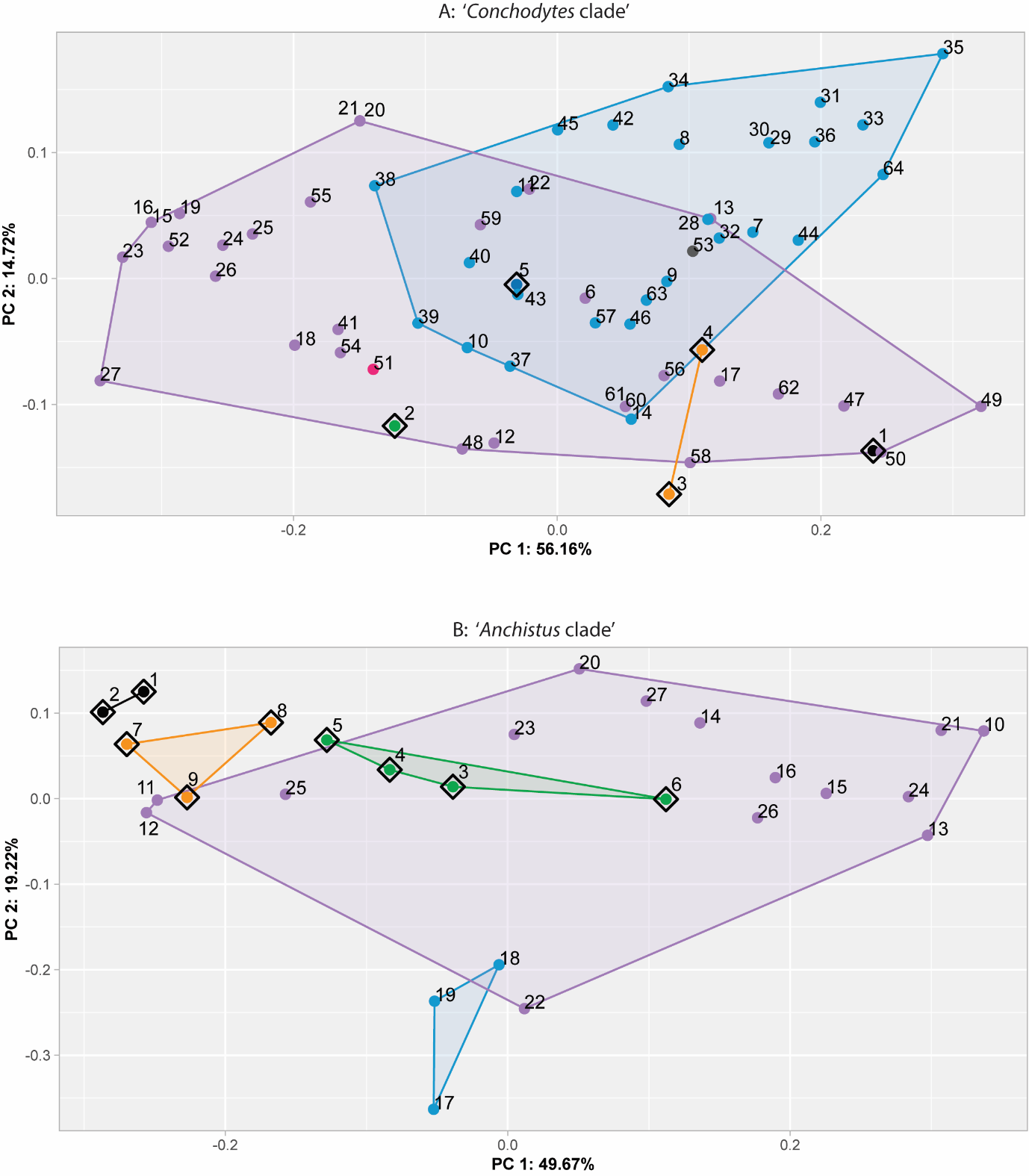


Figure S3 Walking leg dactyli morphospace plots with datapoint numbers.

The plots from the morphospace analyses (2^nd^ and 3^th^ principal component), focussing on the walking leg dactyli morphology, are projected with datapoint numbers below. Numbers correspond with the numbers given in Table S1 (column: ‘Nr.’). Colours corresponds to host choices as in the figures in the main text.


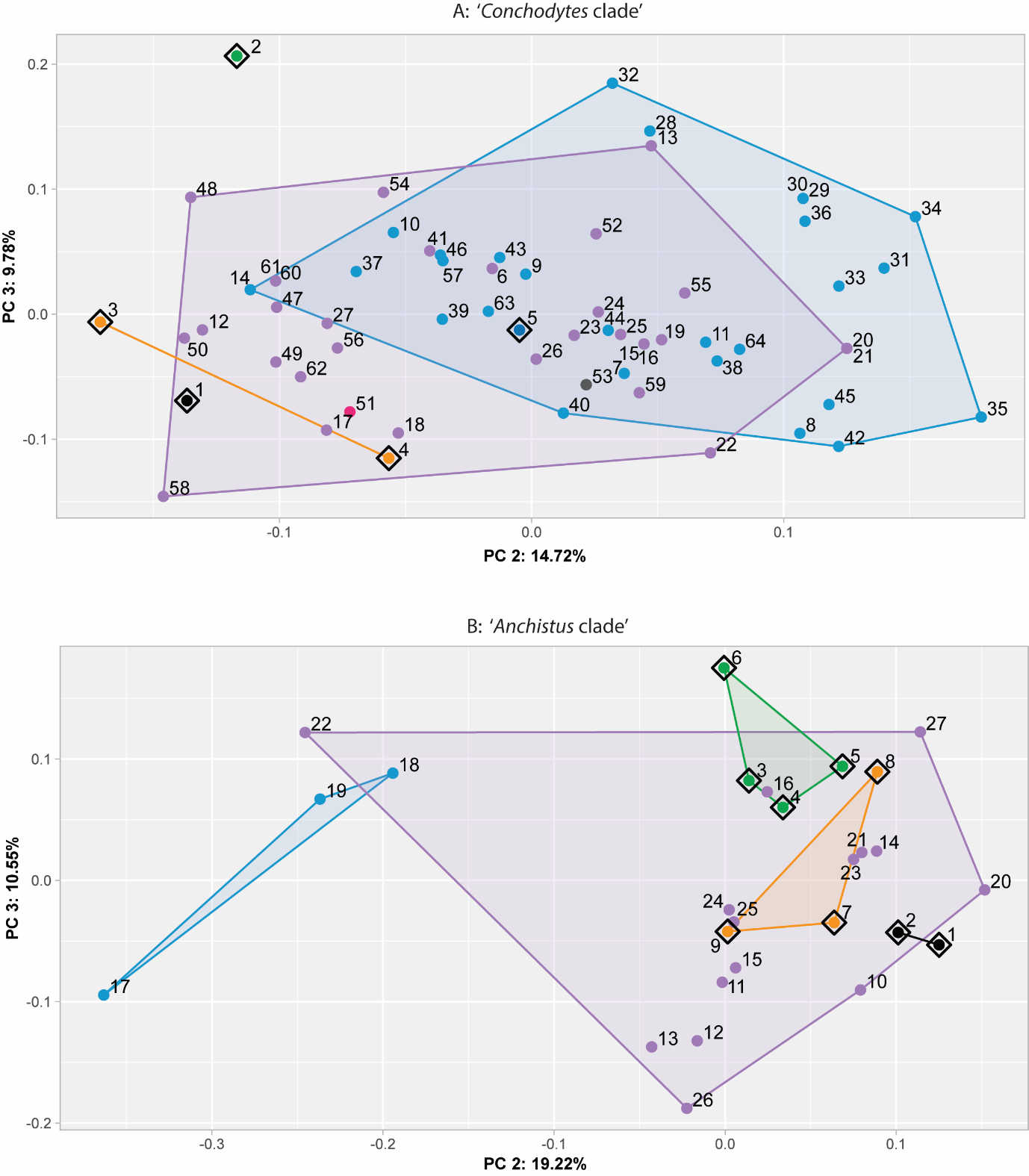
­­

Figure S4 Walking leg dactyli morphospace plots with datapoint numbers.

The plots from the morphospace analyses (1^st^ and 2^nd^ principal component in a, and 2^nd^ and 3^rd^ in b), focussing on the walking leg dactyli morphology, are projected with datapoint numbers below. Numbers correspond with the numbers given in Table S1 (column: ‘Nr.’, within brackets). Colours corresponds to host choices as in the figures in the main text.


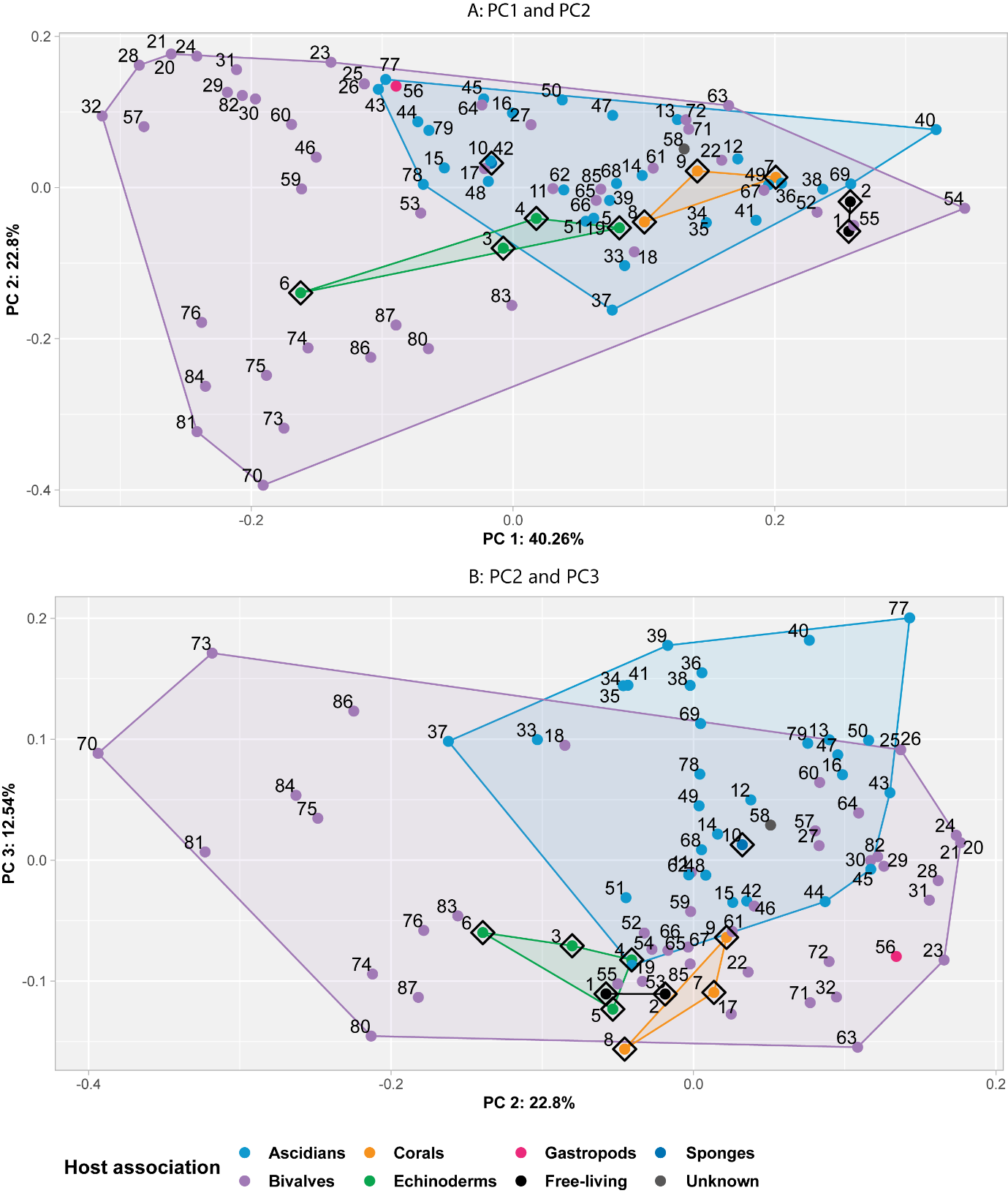


Figure S5 SEM captures of third pair of pereiopod dactyli of species within the ‘*Conchodytes* clade’ (1/4), with detailed insets.

Genera *Conchodytes* and *Platypontonia*. a: *Platypontonia hyotis* Hipeau-Jacquotte, 1971; b: *Conchodytes nipponensis* (De Haan, 1844); c: *C. maculatus* Bruce, 1989; d: *C. tridacnae* Peters, 1852; e: *C. meleagrinae* Peters, 1852; f: *C.* *biunguiculatus* (Paulson, 1875); g: *C. pteriae* Fransen, 1994; h: *C. monodactylus* Holthuis, 1952; i: *C. chadi* (Marin, 2011); j: *C. placunae* (D. S. Johnson, 1967). Scale bars: a: 200 µm; b–f, h–j: 100 µm; g: 50 µm.

**
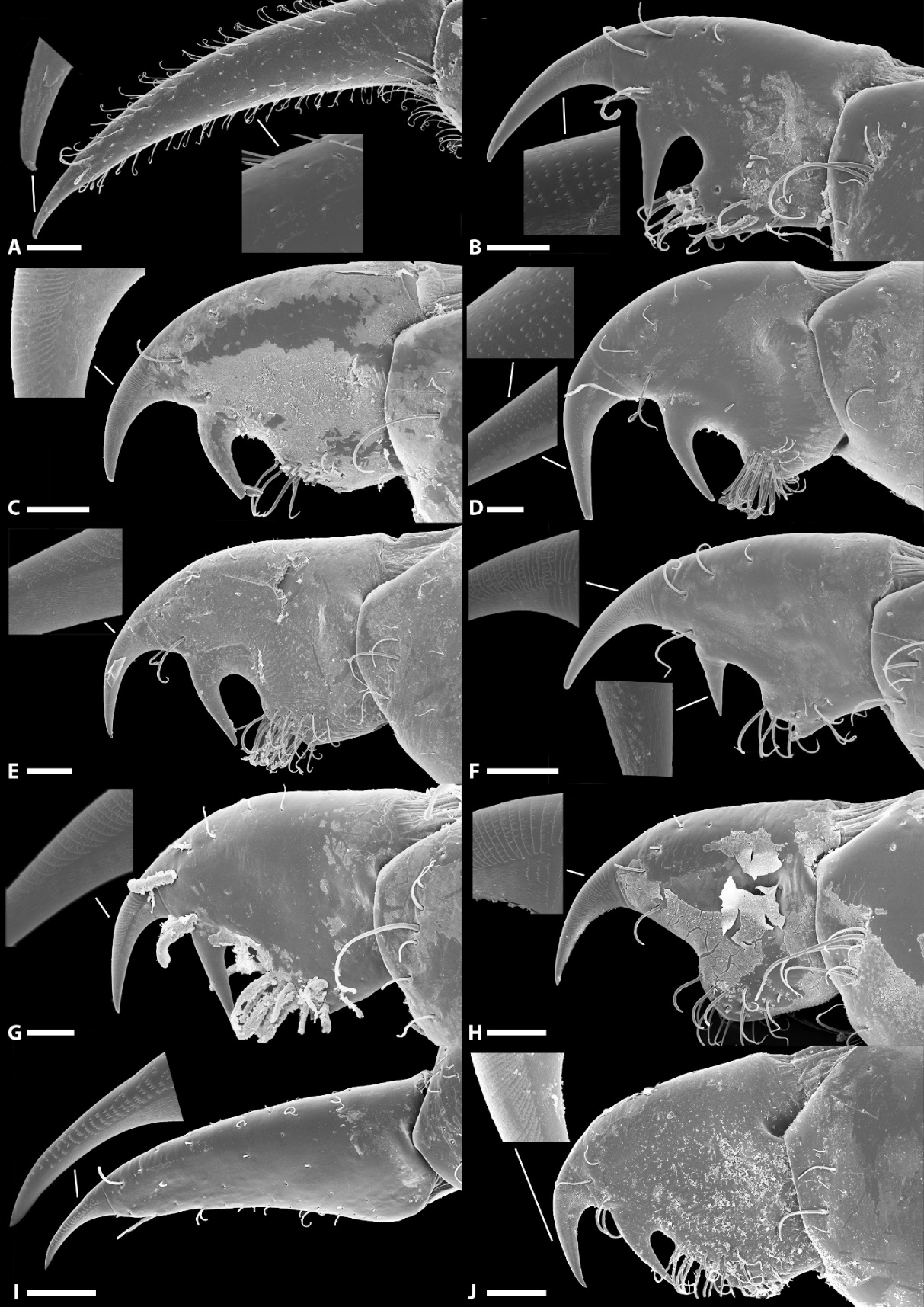
**

Figure S6 SEM captures of third pair of pereiopod dactyli of species within the ‘*Conchodytes* clade’ (2/4), with detailed insets.

Genera *Anchiopontonia* and *Odontonia*. a: *Anchiopontonia* *hurii* (Holthuis, 1981), with part of the underside of the unguis showing in the inset; b: *Odontonia* *rufopunctata* Fransen, 2002; c: *O. sibogae* (Bruce, 1973); d: *O. bagginsi* De Gier & Fransen, 2018; e: *O. katoi* (Kubo, 1940); f: *O. seychellensis* Fransen, 2002; g: *O. plurellicola* De Gier & Fransen, 2018. Scale bars: a: 100 µm; b: 20 µm; c–g: 50 µm. Figures of *Odontonia* after de Gier & Fransen (2018).


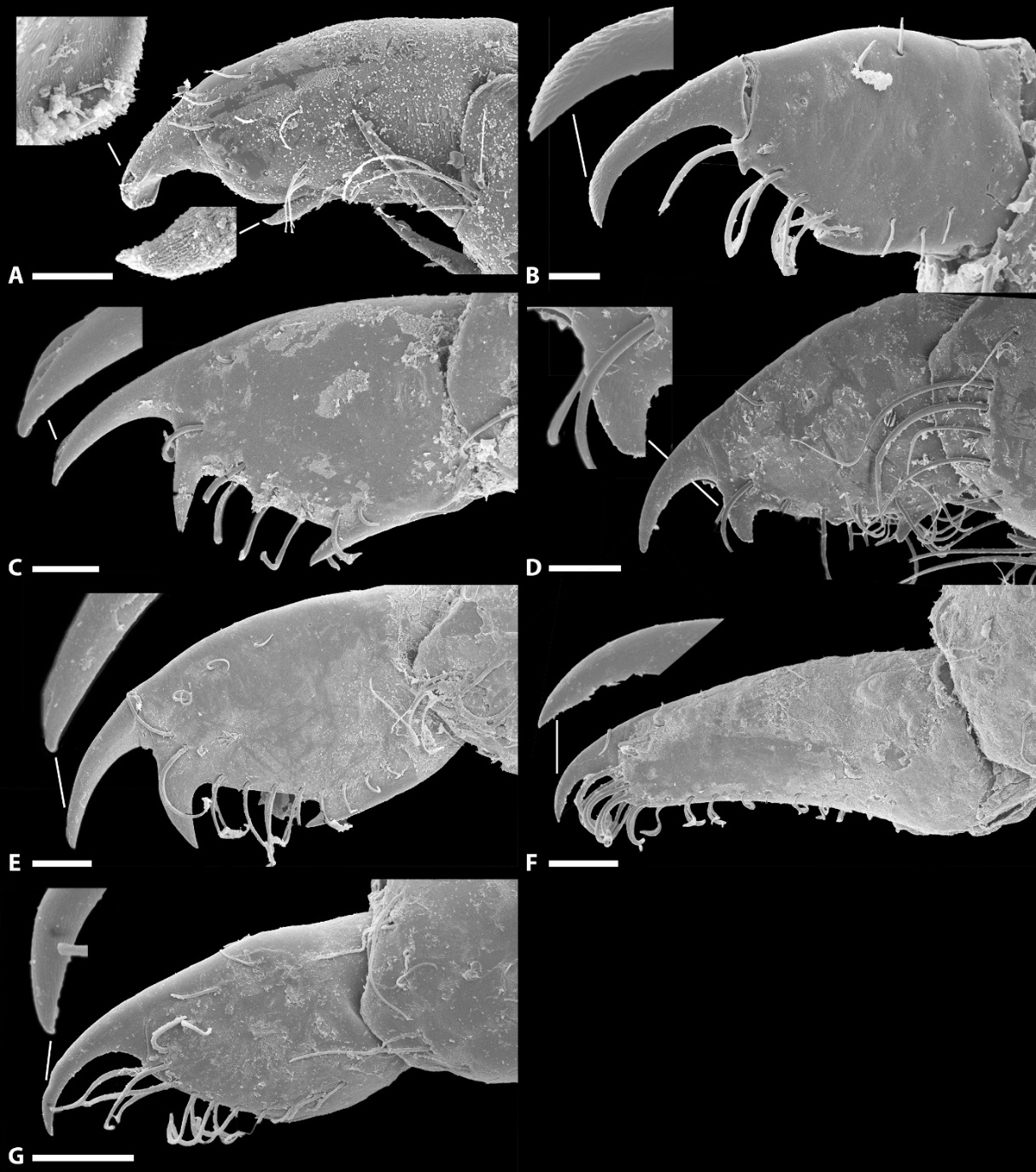


Figure S7 SEM captures of third pair of pereiopod dactyli of species within the ‘*Conchodytes* clade’ (3/4), with detailed insets.

Genera *Ascidonia* and *Pontonia*. a: *Ascidonia flavomaculata* (Heller, 1864); b: *Ascidonia* *quasipusilla* (Chace, 1972); c: *Pontonia manningi* Fransen, 2000; d: *P. margarita* Smith in Verrill, 1869; e: *P. pinnophylax* (Otto, 1821); f: *P.* *mexicana* Guérin-Méneville, 1855; g: *P. pinnae* Lockington, 1878. Scale bars: a: 50 µm; b–g: 100 µm.

**
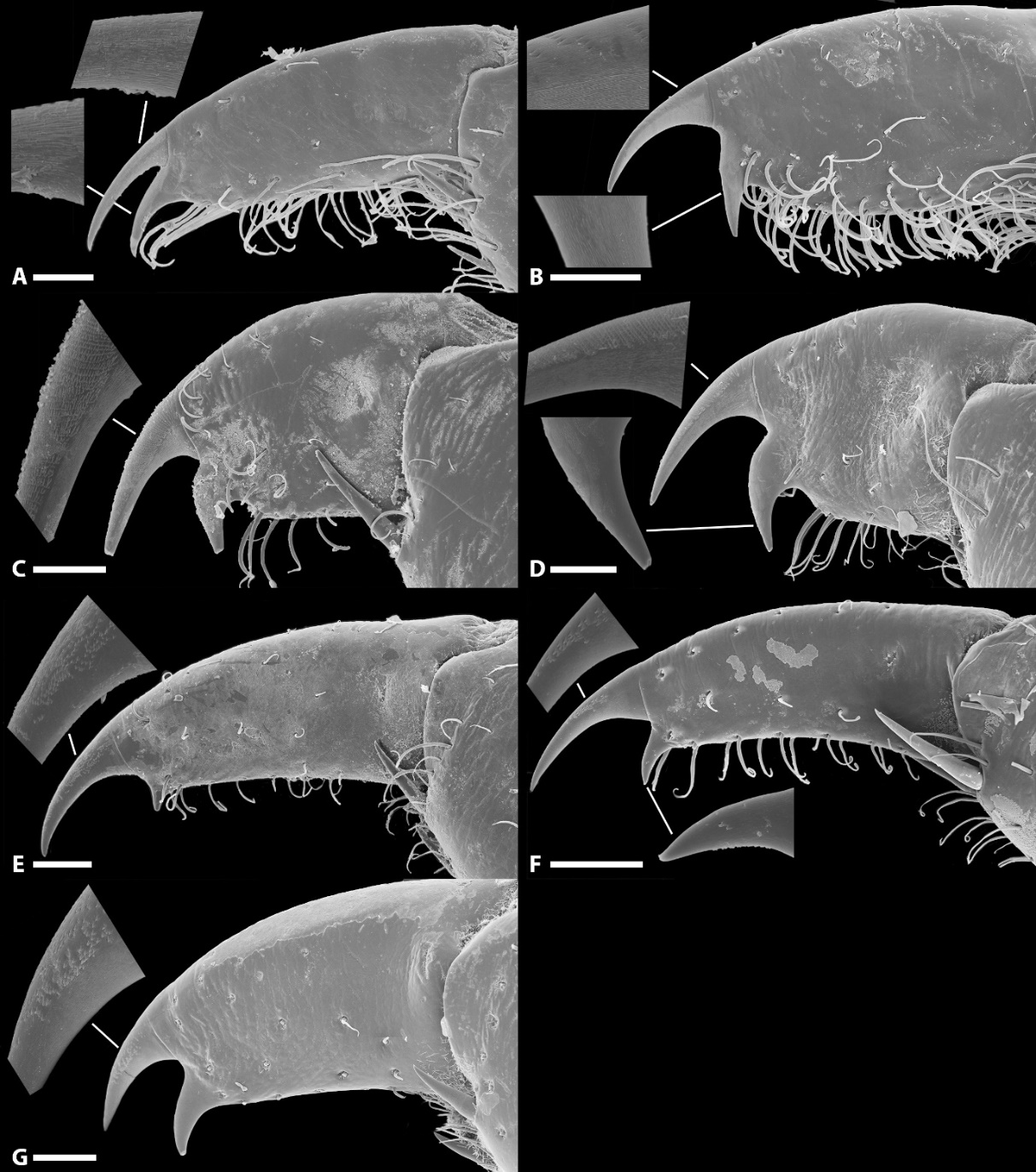
**

Figure S8 SEM captures of third pair of pereiopod dactyli of species within the ‘*Conchodytes* clade’ (4/4), with detailed insets.

Genera *Dactylonia* and *Rostronia*. a: *Rostronia stylirostris* (Holthuis, 1952); b: *Dactylonia* *okai* (Kemp, 1922); c: *D. holthuisi* Fransen, 2002; d: *D. ascidicola* (Borradaile, 1898); e: *D. anachoreta* (Kemp, 1922). Scale bars: a–c, e: 50 µm; d: 100 µm.


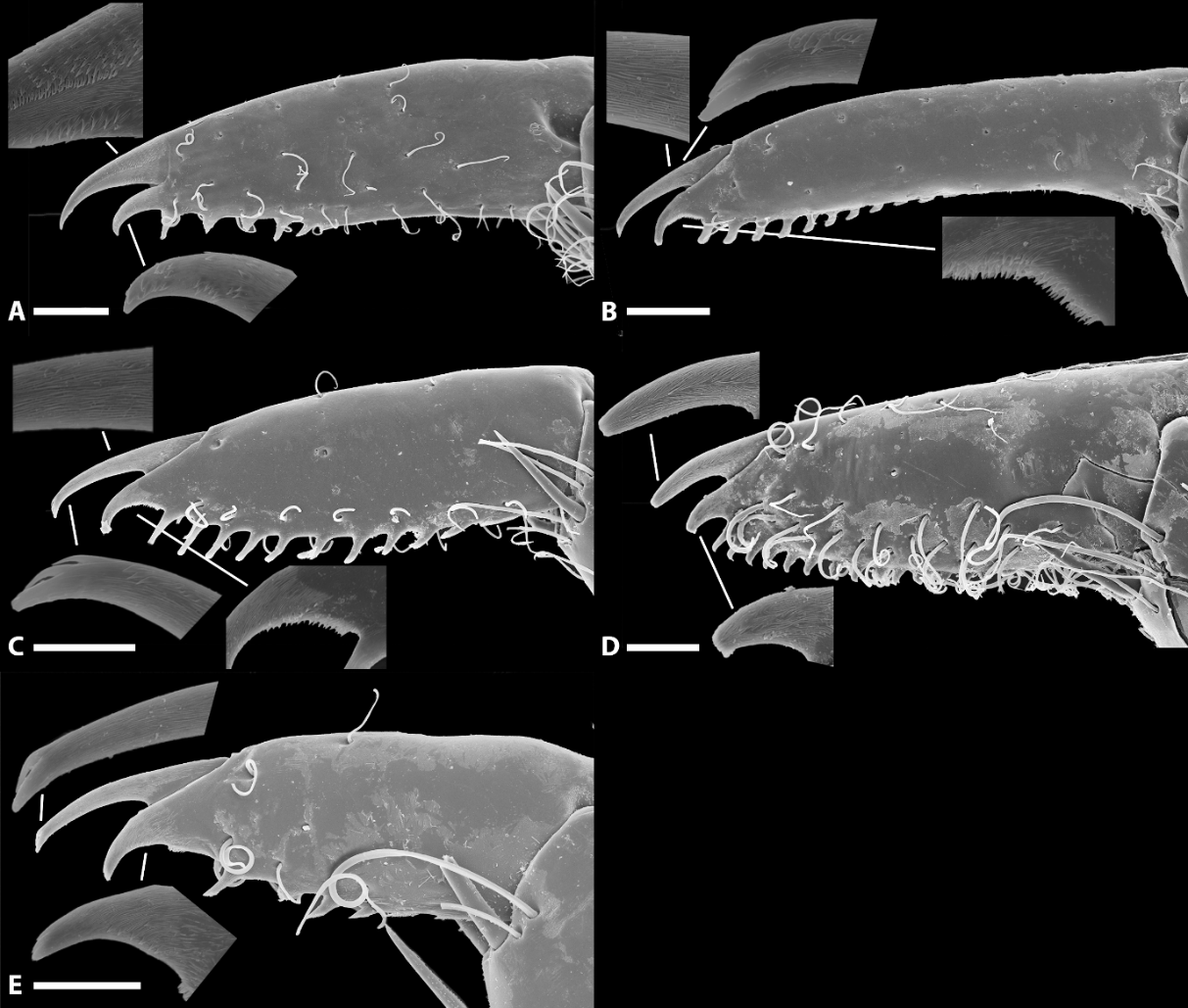


Figure S9 SEM captures of third pair of pereiopod dactyli of species within the ‘*Anchistus* clade’ (1/2), with detailed insets.

Genera *Dasella*, *Ensiger*, *Paranchistus*, and *Polkamenes*. a: *Dasella* *herdmaniae* (Lebour, 1938); b: *Paranchistus* *armatus* (H. Milne Edwards, 1837); c: *Ensiger* *custos* (Forskål, 1775); d: *E.* *custoides* (Bruce, 1977); e: *Polkamenes* *pycnodontae* (Bruce, 1978) (hosted by *Hyotissa* *hyotis* (L., 1758)); f: *P.* *pycnodontae* (hosted by *Spondylus* sp.). Scale bars: a–d: 100 µm; e, f: 50 µm.

**
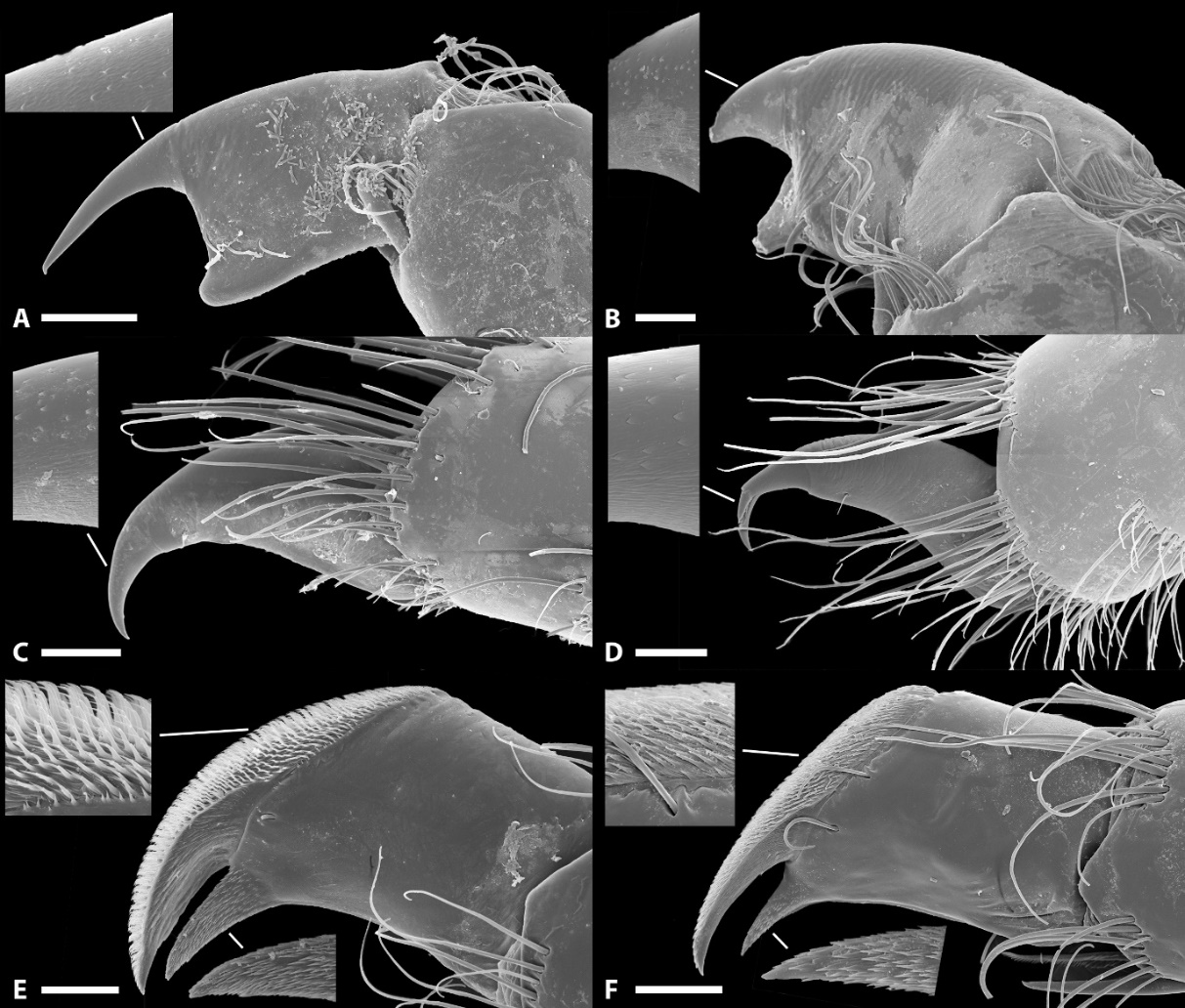
**

Figure S10 SEM captures of third pair of pereiopod dactyli of species within the ‘*Anchistus* clade’ (2/2), with detailed insets.

Genera *Anchistus*, *Neoanchistus*, and *Tympanicheles*. a: *Tympanicheles ornatus* (Holthuis, 1952); b: *T. pectinis* (Kemp, 1925); c: *Neoanchistus nasalis* Holthuis, 1986; d: *Anchistus australis* Bruce, 1977; e: *A. miersi* (De Man, 1888); f: *A. demani* Kemp, 1922. Scale bars: a–c, e, f: 50 µm; d: 100 µm.

**
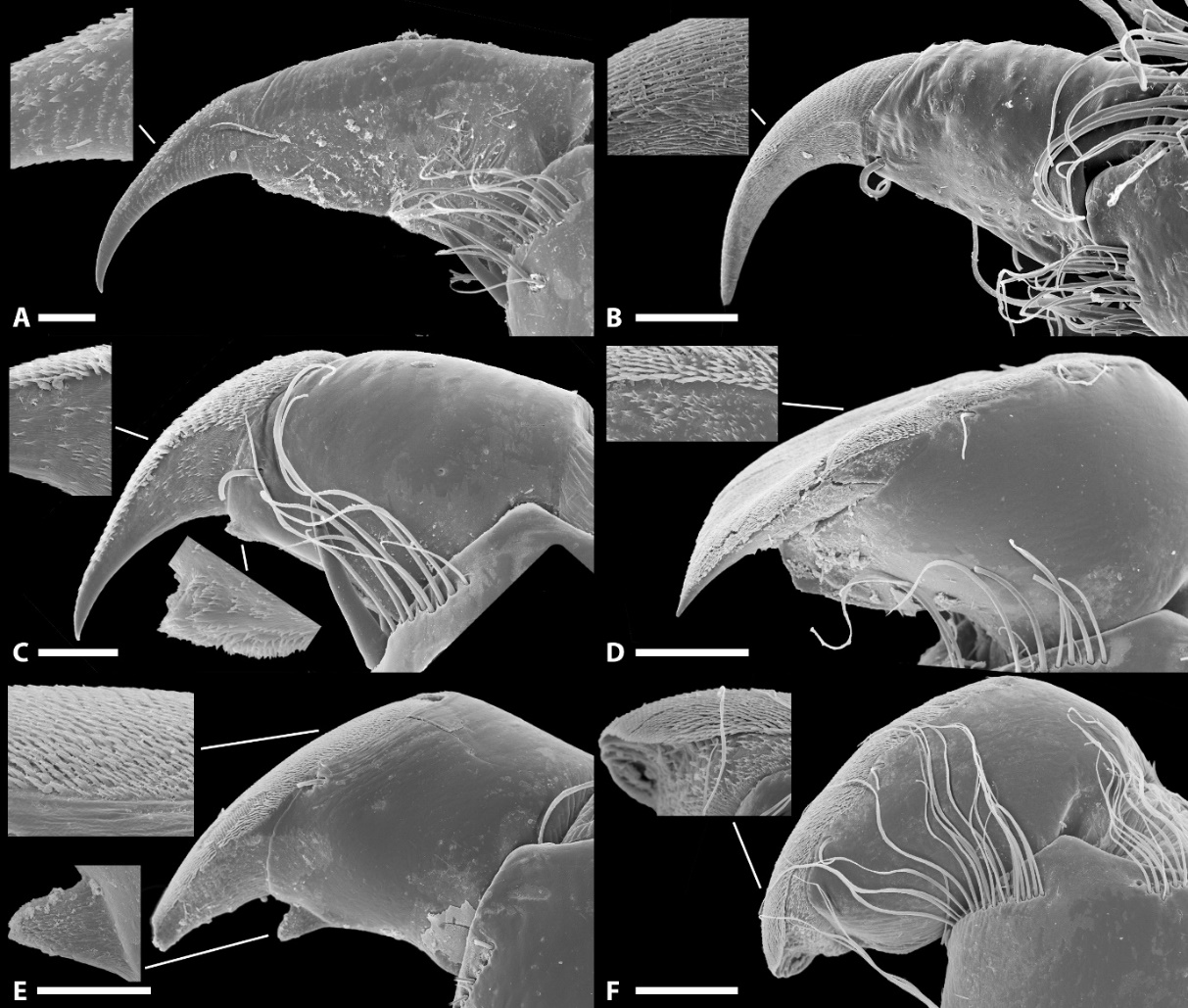
**

Figure S11 SEM captures of third pair of pereiopod dactyli of species within the outgroup, with detailed insets.

a: *Typton wasini* Bruce, 1977; b: *Zenopontonia rex* (Kemp, 1922); c: *Z. soror* (Nobili, 1904); d: *Lipkemenes* *lanipes* (Kemp, 1922); e: *Actinimenes inornatus* (Kemp, 1922); f: *Periclimenes kempi* Bruce, 1969; g: *Cuapetes tenuipes* (Borradaile, 1898); h: *Palaemonella rotumana* (Borradaile, 1898). Scale bars: a, c–d: 20 µm; b, e–f: 50 µm; g: 200 µm; h: 100 µm.


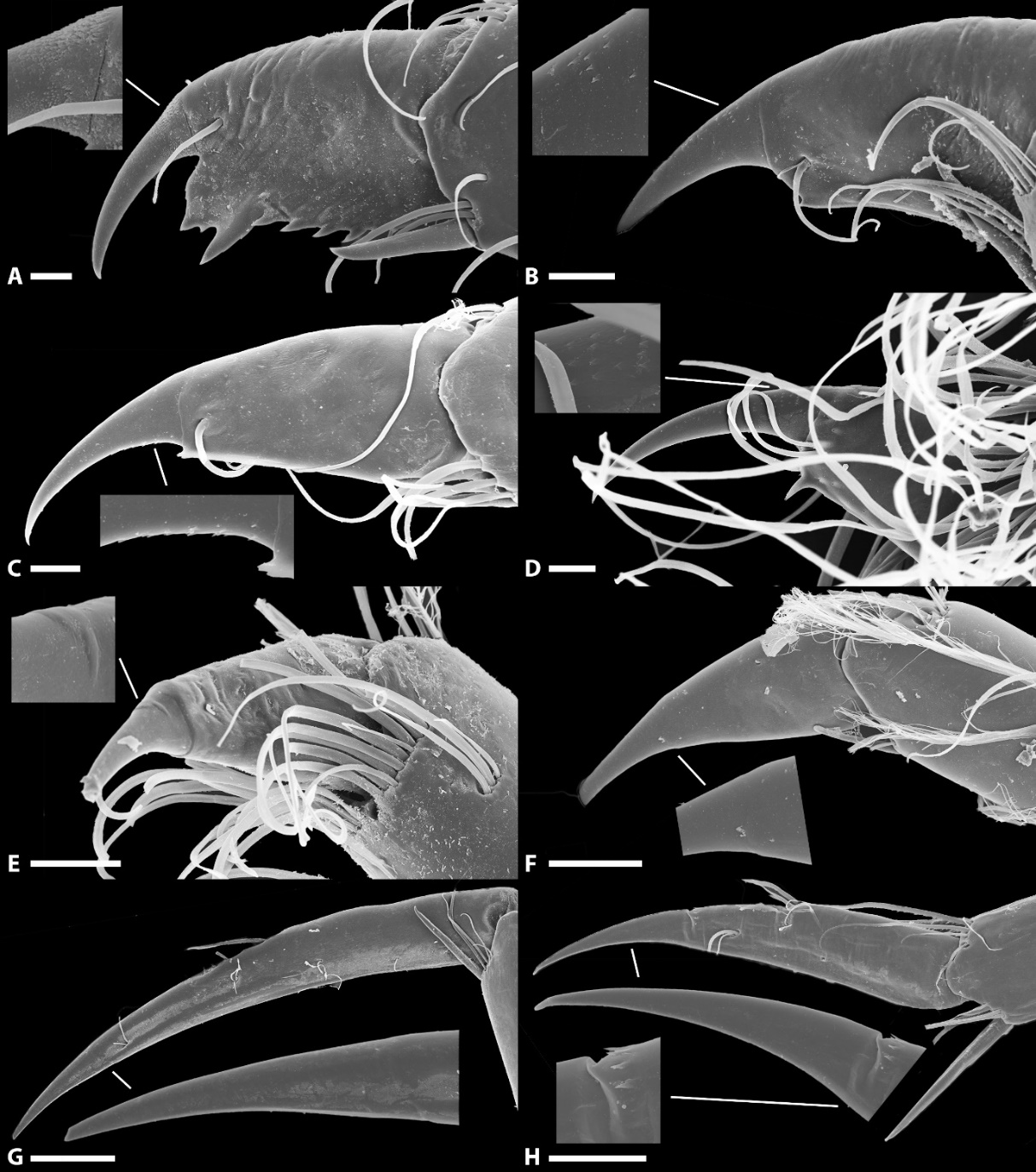

Supplement: Supplementary file 1 — Appendix S1. [file ECE3-13-e10768-s001.docx]
